# Cytotoxicity screening of 23 engineered nanomaterials using a test matrix of ten cell lines and three different assays

**DOI:** 10.1186/1743-8977-8-9

**Published:** 2011-02-23

**Authors:** Alexandra Kroll, Christian Dierker, Christina Rommel, Daniela Hahn, Wendel Wohlleben, Christian Schulze-Isfort, Christian Göbbert, Matthias Voetz, Ferdinand Hardinghaus, Jürgen Schnekenburger

**Affiliations:** 1 Biomedizinisches Technologiezentrum, Westfälische Wilhelms-Universität, Domagkstraße 3a, 48149 Münster, Germany; 2 BASF SE, Polymer Physics Research, Ludwigshafen, Germany; 3 Evonik Degussa GmbH, Inorganic Materials, Hanau, Germany; 4 CWT Clean Tec AG, Halberstadt, Germany; 5 Bayer Technology Services, Leverkusen, Germany; 6 Solvay Infra Bad Hönningen GmbH, Bad Hönningen, Germany; 7 Eawag, ETH Domain, Überlandstrasse 133, 8600 Dübendorf, Switzerland

## Abstract

**Background:**

Engineered nanomaterials display unique properties that may have impact on human health, and thus require a reliable evaluation of their potential toxicity. Here, we performed a standardized *in vitro *screening of 23 engineered nanomaterials. We thoroughly characterized the physicochemical properties of the nanomaterials and adapted three classical *in vitro *toxicity assays to eliminate nanomaterial interference. Nanomaterial toxicity was assessed in ten representative cell lines.

**Results:**

Six nanomaterials induced oxidative cell stress while only a single nanomaterial reduced cellular metabolic activity and none of the particles affected cell viability. Results from heterogeneous and chemically identical particles suggested that surface chemistry, surface coating and chemical composition are likely determinants of nanomaterial toxicity. Individual cell lines differed significantly in their response, dependent on the particle type and the toxicity endpoint measured.

**Conclusion:**

*In vitro *toxicity of the analyzed engineered nanomaterials cannot be attributed to a defined physicochemical property. Therefore, the accurate identification of nanomaterial cytotoxicity requires a matrix based on a set of sensitive cell lines and *in vitro *assays measuring different cytotoxicity endpoints.

## Background

Engineered nanomaterials (NMs) are used in various industrial applications including cosmetics, electronics and coatings, as well as automobile technology. Their increasing production and the possible risk of human exposure to these nanomaterials have prompted the need for a detailed understanding of the potential toxicity. Due to their unique size-related characteristics, NMs seem to display biological effects in animal models and in cultured cells that differ from those observed with bulk material. For instance, TiO_2 _NMs frequently used in sunscreens and paints have been shown to induce a much greater pulmonary inflammatory response in rats as compared to fine-sized particles of the same chemical composition [1-3] and ROS induction in lung epithelial cells exposed to TiO_2 _NMs was greater than that of fine-sized TiO_2 _particles [4]. Physicochemical properties of NMs as crystallinity [5,6], agglomeration state [7] and solubility [8] have been associated with the toxic potential. However, the available data do not allow identifying specific NM properties that are responsible for NM toxicity. Considering the huge number of potential NM variables that may determine the biological impact, each new NM type has to be tested individually and characterized regarding its physicochemical properties. This requires integrated testing strategies minimizing the need for animal studies. Currently, standardized *in vitro *tests and experimental protocols suitable for NM toxicity testing are not available. Recent studies demonstrate that classic cytotoxicity assays may not be suitable to assess NM toxicity since NMs can interfere with assay reagents or detection systems thereby generating false positive/negative results [9-11].

Due to the lack of standards for NM testing, current data on NM toxicity are often inconsistent and can hardly be compared. Adverse effects on cells have been observed mostly with NM concentrations much higher than tissue concentrations in animal models. The relevancy of these results is questionable [12,13] and current comparisons of *in vitro *vs. *in vivo *studies found little correlation [14,15]. This underlines the urgent need for standardized NM cytotoxicity assessment, which requires carefully evaluated *in vitro *methodologies. Since the experimental setup has been shown to influence the toxicological outcome (e.g. [16]) standardized protocols for the preparation and characterization of NM dispersions, exposure conditions and reference material have to be developed to improve the comparability between *in vivo *and *in vitro *studies. Recent findings suggest that multiple *in vitro *assays should be employed in nanomaterial toxicity assessments to be able to detect the potentially pleiotropic responses of cells exposed to NMs, but most of the NM toxicity screening approaches published so far were based on single cell lines [10,17]. Furthermore, L'Azou et al. 2008 reported that two different renal cell types exhibited different sensitivity to TiO_2 _NMs indicating that the choice of cell model influences the findings [12].

In this study, which was part of the German national lead project NanoCare [18], we have evaluated and standardized common *in vitro *assays measuring three different cytotoxic endpoints (oxidative stress, metabolic activity, cell death), and adapted them for the toxicity assessment of 23 engineered NMs. Physicochemical, optical and catalytic properties of the NM dispersions were thoroughly characterized, and experimental procedures were modified to exclude particle interference. The NMs represented metal oxides, -sulfates and -carbonates utilized for a wide range of industrial products and applications. Carbon Black was used as reference. To identify NM properties, which are most influential in driving NM toxicity, we analyzed NMs of the same production process or the same chemical composition varying in single or multiple physicochemical properties. We established a matrix based on three standardized *in vitro *assays and ten selected cell lines and assessed the potential toxicity of the 23 different NM types at three different doses.

## Results and Discussion

### Particle Characterization

Nano-specific properties such as particle size and surface area contribute largely to NM toxicity (for review see [13,19]). We therefore thoroughly characterized previously defined essential physicochemical properties of 22 representative engineered NMs and Carbon Black as reference NM [20]. The nanomaterials were selected during the set up of the German project NanoCare. The rational was to use industrial material which is produced for industrial applications. The materials were chosen from the available NM of the participating manufacturers and for the comparison of similar or related chemical compositions. Primary particle size and particle morphology were characterized by monolayer TEM. Particle surface chemistry was determined by photoelectron spectroscopy (XPS) and crystallinity by X-ray diffraction (XRD). Impurities were detected by inductively-coupled-plasma mass-spectrometry (ICP-MS). ζ-potential was assessed by phase analysis light scattering (PALS; Zetasizer Nano ZS, Malvern Instruments) and particle surface by Brunauer-Emmet-Teller analysis (BET) [20,21]. NM solubility in cell culture medium (DMEM/10% FBS) was measured by inductively coupled plasma optical emission spectrometry (ICP-OES). The pH and ζ-potentials were determined both in H_2_O and in DMEM/10% FBS. The results are listed in Table 1 and exemplary TEM images are provided in Figure S1 (Additional file 1). The primary particle size ranged from 10 nm to 250 nm and the surface area from 8.9 m^2^g^-1 ^to 339 m^2^g^-1^. Most NMs were more than 99% pure. The ζ-potentials of the different NM dispersions in DMEM/10% FBS were similar to that of pure DMEM/10% FBS (-7.6 ± 0.9 mV) while the ζ-potentials in H_2_O ranged from -66 mV to +59 mV. The pH of DMEM/10% FBS (7.8) was not influenced by the highest concentration of NMs used (32 μg ml^-1^). All NM dispersions were sterilized and tested to be free of endotoxins.

**Table 1:** Physical and chemical characteristics of the NMs used in the present study

Material	Primary particle size distribution [nm]	BET **[m^2 ^g**^**-1**^**]**	Crystallinity	Particle morphology	Purity [%]	Surface modification	Surface chemistry [%]	ζ - potential **H**_**2**_**O (pH 7)** [mV]	**pH in H**_**2**_**0** (pH 7)	ζ-potential DMEM/10% FBS [mV]	pH DMEM/ 10% FBS	Solubility DMEM/10% FBS **[mg kg**^**-1**^**]**
**TiO**_**2 **_**1**	d_90 _17 d_50 _11	55	anatase (95%)/rutile (5%)	irregular spherical	>99.0	polyoxa acid	O 53.0 Ti 21.0 C 25.0	17.5	4.04	-6.7 ± 0.7	7.8	Ti: 0.063 ± 0.042

**TiO**_**2 **_**2**	d_90 _17 d_50 _11	55	anatase (95%)/rutile (5%)	irregular spherical	>99.0	polyoxa acid	O 56.4 Ti 20.1 C 22.8	17.5	4.04	-9.9 ± 0.6	7.8	Ti: 0.073 ± 0.024

**TiO**_**2 **_**3**	d_90 _28 d_50 _21 25*	51	anatase (80%)/rutile (20%)	crystalline, irregular globular	99.5	-	0 58.0 Ti 26.0 C 14.0 N 0.5 Cl 1.0	-26.3	3.62	-9.1 ± 0.3	7.8	Ti: 0.015 ± 0.004

**Carbon Black**	d_90 _23 d_50 _17 14*	339	amorphous	agglomerated (d_90 _37 μm; d_50 _127 μm)	> 99.9	-	nd	-14	nd	-7.6 ± 0.9	7.8	nd

**CeO**_**2**_**-A**	d_90 _10 d_50 _7	63	100% face centered cubic	crystalline	> 99.97	HNO_3_	O 56.8 Ce 25.0 C 18.2	45.8	6.2	-9.9 ± 0.5	7.8	Ce: 0.415 ± 0.005

**CeO**_**2**_**-B**	d_90 _15 d_50 _7	44	100% face centered cubic	crystalline	> 99.97	HNO_3_	O 56.1 Ce 21.8 C 22.1	35	5.4	-9.2 ± 0.2	7.8	Ce: 0.57 ± 0.08

**CeO**_**2**_**-C**	d_90 _16 d_50 _7	38	100% face centered cubic	crystalline	> 99.97	HNO_3_	O 56.5 Ce 22.1 C 21.5	30	3.4	-10.1 ± 0.2	7.8	Ce: 0.255 ± 0.040

**CeO**_**2**_**-D**	d_90 _10 d_50 _7	63	100% face centered cubic	crystalline	> 99.97	HNO_3_	O 56.0 Ce 22.3 C 21.7	30	3.4	-9.6 ± 0.1	7.8	Ce: 0.28 ± 0.06

**CeO**_**2**_	d_90 _70 d_50 _40	33	cerianite, cubic	irregular spherical	97.7	-	O 53.0 Ce 26.0 C 20.0 Cl 0.6	23.7	nd	-7.3 ± 1.1	7.9	Ce: 0.018 ± 0.016

**AlOOH I**	d_50 _40 d_90 _nd	47	orthorhombic, Böhmit-like	irregular spherical, agglomerated (d_50 _41 μm; d_90 _83 μm)	82.7	-	O 61.6 Al 31.7 C 6.7	34.0 (1 mmol KCl)	3.5-5	-7.6 ± 0.8	7.8	Al: 0.185 ± 0.012

**AlOOH II**	d_50 _70 d_90 _nd	159	orthorhombic, Böhmit-like	irregular spherical, agglomerated (d_50 _12 μm; d_90 _43 μm)	92.5	-	O 65.0 Al 32.1 C 2.0 Cl 0.9	26.4 (1 mmol KCl)	7.5	-10.7 ± 0.2	7.8	Al: 0.265 ± 0.167

Ti-Zr 1*	d_90 _31 d_50 _23	44	ZrO_2 _tetragonal/baddeleyite ZrO_2_	crystalline	>99 TiO_2_:ZrO_2 _= 10:90	-	C 16.9 O 55.5 Cl 0.7 Ti 6.3 Zr 20.6	-30	4.0	-8.7 ± 0.7	7.8	Ti: - Zr: 0.012 ± 0.001

Ti-Zr 2*	d_90 _34 d_50 _17	54	ZrO_2 _srilankite orthorhombic, TiO_2 _possibly rutile	crystalline	>99 TiO_2_:ZrO_2 _= 50:50	-	C 20.5 O 55.3 Cl 0.4 Ti 15.6 Zr 10.2	-34	4.1	-9.7 ± 0.4	7.8	Ti: 0.011 ± 0.007 Zr: 0.018 ± 0.006

Ti-Zr 3*	d_90 _34 d_50 _18	51	TiO_2 _tetragonal anatase and rutile	crystalline	>99 TiO_2_:ZrO_2 _= 90:10	-	C 22.6 O 53.7 Ti 22.4 Zr 1.2	-20	4.4	-8.9 ± 0.3	7.8	Ti: 0.028 ± 0.002 Zr: 0.004 ± 0.002

Al-Ti-Zr 1*	d_90 _30 d_50 _23	43	ZrO_2 _tetragonal	31% ellipsoid 36% linear 31% branched 2% circular	>99 Al_2_O_3_:ZrO_2 _= 15:75 TiO_2 _10	-	C 13.5 O 56 Al 14.2 Cl 0.9 Ti 5.8 Zr 9.6	0	4.61	-9.5 ± 0.7	7.8	Al: 0.085 ± 0.046 Ti: 0.011 ± 0.007 Zr: 0.030 ± 0.005

Al-Ti-Zr 2*	d_90 _36 d_50 _25	45	AlO_0,18_ZrO_0,82_O_1,91_tetragonal ZrO_2 _orthorhombic possibly TiO_2 _cubic	36% ellipsoid 37% linear 24% branched 3% circular	>99 Al_2_O_3_:ZrO_2 _= 45:45 TiO_2 _10	-	C 12.1 O 57 Al 20.9 Cl 0.7 Ti 3.2 Zr 6.1	18	4.93	-7.4 ± 0.7	7.8	Al: 0.045 ± 0.012 Ti: 0.008 ± 0.004 Zr: 0.029 ± 0.018

Al-Ti-Zr 3*	d_90 _41 d_50 _29	48	AlO_0,18_ZrO_0,82_O_1,91_tetragonal Al_2_O_3 _cubic	36% ellipsoid 33% linear 27% branched 3% circular	>99 Al_2_O_3_:ZrO_2 _= 75:15 TiO_2 _10	-	C 13.5 O 54.4 Al 23.9 Cl 0.4 Ti 4.6 Zr 3.3	19	4.57	-7.0 ± 0.5	7.8	Al: 0.01 ± 0.018 Ti: 0.003 ± 0.004 Zr: 0.008 ± 0.001

**ZrO**_**2 **_**1**	d_90 _40 d_50 _17	122	ZrO_2 _monocline (60%)/Baddeleyite tetragonal (40%)	irregular spherical	94.6	polyoxa acid, 12,5% w/w	O 55 Zr 21 C 24 Cl 0.6	59.3	4.06	-8.7 ± 0.8	7.8	Zr: 0.037 ± 0.032

**ZrO**_**2 **_**2**	d_90 _40 d_50 _17	122	ZrO_2 _monocline (60%)/Baddeleyite tetragonal (40%)	irregular spherical	94.6	acetic acid, 8,5% w/w	O 55 Zr 21 C 24 Cl 0.6	17	3.68	-7.7 ± 0.5	7.8	Zr: 0.062 ± 0.023

**ZrO**_**2 **_**3**	d_90 _250 d_50 _90	96	ZrO_2 _monocline (60%)/Baddeleyite tetragonal (40%)	irregular spherical	94.6	Ammoniumpolyacrylate, 13,6% w/w	O 48 Zr 21 C 18 N 12 Cl 0.6	-44	9.45	-11.5 ± 0.7	7.8	Zr: 0.214 ± 0.086

**BaSO**_**4**_	d_50 _37.5 d_90 _nd	41.4	crystalline, orthorhombic, baryte-like	spherical. agglomerated (d_90 _35 μm; d_50 _17 μm)	93.8	organic	Ba 12.7 S 10.8 O 51.7 C 17.2	-33	9.7	-10.7 ± 0.8	7.8	Ba: 0.675 ± 0.075

**SrCO**_**3 **_**1**	d_90 _19 d_50 _13	33	crystalline, orthorhombic, strontianite-like	rod shaped. agglomerated (d_90 _23.7 μm; d_50 _5.9 μm)	96.4	organic, hydrophilic	Sr 21.1 C 27.1 O 51.3	-10	7.8	-9.2 ± 0.6	7.8	Sr: 0.545 ± 0.092

**SrCO**_**3 **_**2**	d_90 _17 d_50 _13	8.9	crystalline, orthorhombic, strontianite-like	rod shaped. agglomerated (d_90 _18.6 μm; d_50 _5.9 μm)	85	organic, hydrophobic	Sr 11.7 C 50.6 O 37.0 P 0.7	-66	7.6	-10.8 ± 0.6	7.8	Sr: 0.680 ± 0.005

• supplier data, ● mixed oxide, ^1 ^ζ-potential DMEM/10% FBS:-7.6 ± 0.9 mV,

The influence of physicochemical properties on particle toxicity was investigated using different particles of the same chemical composition. For instance, TiO_2 _1 and 2 NMs represent identical particle preparations except that TiO_2 _2 was predispersed at pH 2 in HNO_3_. Both particles were modified with organic polyoxa acid. In contrast, TiO_2 _3 was naked, had a slightly larger particle size, smaller surface area and contained less anatase. Moreover, we have used chemically identical NMs generated by varying parameters in the same production line to yield slightly different primary particle sizes and surface chemistry (CeO_2 _NMs A to D). Please take note of CeO_2_-A and -D that were identical particles regarding size and surface area, but varied slightly in their surface chemistry. CeO_2_-B and -C NMs were slightly larger and had a smaller surface area than CeO_2_-A and -D. The surface modification of CeO_2_-A to -D NMs was identical as was crystallinity, crystal morphology and shape (Table 1). We also included CeO_2 _NMs from a different supplier that varied significantly from CeO_2_-A to -D NMs regarding size, crystal morphology, shape and surface modification (CeO_2_, Table 1).

To determine the influence of the chemical composition on particle toxicity, we used mixed oxide NMs with similar physical properties. For instance, Ti-Zr 1 to 3 NMs had similar primary particle sizes and surface areas but contained increasing ratios of TiO_2 _to ZrO_2 _with Ti-Zr 1 NMs consisting of 10% TiO_2 _and Ti-Zr 3 NMs consisting of approx. 90% TiO_2 _(Table 1). The Al-Ti-Zr mixed oxide NMs 1 to 3 were of similar size and surface area but contained different ratios of ZrO_2_, Al_2_O_3 _and TiO_2 _(Table 1).

The influence of surface modifications on particle toxicity was investigated with NMs of the same production process but with different surface modifications (ZrO_2 _NMs 1 to 3).

### Test procedure standardization

We have chosen three commonly used *in vitro *test systems monitoring different cytotoxicity endpoints: oxidative stress (DCF assay), cell death (LDH assay) and cellular metabolic activity (MTT assay). The DCF assay monitors the formation of intracellular ROS via a fluorescent product (DCF) generated by the oxidation of the non-fluorescent substrate H_2_DCF-DA. In contrast, MTT and LDH assays are colorimetric assays based on the reduction of light-absorbing substrates (MTT and INT, respectively). While MTT is reduced by metabolically active cells, INT was used as a substrate for LDH released by damaged cells. Since NM interference with *in vitro *test systems has been consistently reported [11], we analyzed the influence of each of the 23 types of NMs on the selected assays.

The intrinsic optical activities were determined for all NM dispersions with particle concentrations of 0.01, 0.1, 1, 10, 50, and 100 μg cm^-2^. To analyze the interference of NM dispersions with the fluorescence measurement of DCF, dilutions of fluorescent DCF covering the full detection range of the spectrofluorometer were measured in medium or in the presence of NM dispersions following the assay protocols (for details see Experimental Section). All NM dispersions interfered with the original protocol for the detection of DCF fluorescence at concentrations of 10 μg cm^-2 ^and above (Table 2). NM interference with MTT light absorption was determined using different mixtures of yellow MTT and purple MTT-formazan that covered the whole spectrum of light absorption from the reactant to the product. Light absorption of MTT was strongly altered in the presence of all nanomaterial dispersions at concentrations of 10 μg cm^-2 ^and above (Table 2). At lower particle concentrations (1 μg cm^-2^), interference could still be detected with Carbon Black, TiO_2 _2, TiO_2 _3 and Al-Ti-Zr 3 (data not shown).

**Table 2:** Interference of nanomaterials with *in vitro *toxicity test systems

	Parameters and particle condition									
	**Oxidative stress**				**Metabolic activity**				**Cell death**	

	**DCF fluorescence**				**MTT light absorption**				**INT light absorption**	

	**Particle dispersion on cells** **[μg cm**^**-2**^**]**		**Particles on cells after washing** **[μg cm**^**-2**^**]**		**Particle dispersion on cells** **[μg cm**^**-2**^**]**		**Particles on cells after washing** **[μg cm**^**-2**^**]**		**Particle dispersions cell free** **[μg cm**^**-2**^**]**	

**Particles**	**10**	**50**	**10**	**50**	**10**	**50**	**10**	**50**	**10**	**50**

**AlOOH I**	**+**	**+**	-	**+**	**+**	**+**	-	**+**	-	**+**

**AlOOH II**	**+**	**+**	-	**+**	**+**	**+**	-	**+**	-	**+**

**Al-Ti-Zr 1**	**+**	**+**	-	**+**	**+**	**+**	-	**+**	-	**+**

**Al-Ti-Zr 2**	**+**	**+**	-	**+**	**+**	**+**	-	**+**	-	**+**

**Al-Ti-Zr 3**	**+**	**+**	-	**+**	**+**	**+**	-	**+**	-	**+**

**BaSO**_**4**_	**+**	**+**	-	**+**	**+**	**+**	-	**+**	-	**+**

**Carbon Black**	**+**	**+**	**(+)**	**+**	**+**	**+**	**+**	**+**	-	**+**

**CeO**_**2**_	**+**	**+**	-	**+**	**+**	**+**	-	**+**	-	**+**

**CeO**_**2 **_**A**	**+**	**+**	-	**+**	**+**	**+**	-	**+**	-	**+**

**CeO**_**2 **_**B**	**+**	**+**	-	**+**	**+**	**+**	-	**+**	-	**+**

**CeO**_**2 **_**C**	**+**	**+**	-	**+**	**+**	**+**	-	**+**	-	**+**

**CeO**_**2 **_**D**	**+**	**+**	-	**+**	**+**	**+**	-	**+**	-	**+**

**SrCO**_**3 **_**1**	**+**	**+**	-	**+**	**+**	**+**	-	**+**	-	**+**

**SrCO**_**3 **_**2**	**+**	**+**	-	**+**	**+**	**+**	-	**+**	-	**+**

**TiO**_**2 **_**2**	**+**	**+**	-	**+**	**+**	**+**	-	**+**	-	**+**

**TiO**_**2 **_**1**	**+**	**+**	-	**+**	**+**	**+**	-	**+**	-	**+**

**TiO**_**2 **_**3**	**+**	**+**	-	**+**	**+**	**+**	-	**+**	-	**+**

**Ti-Zr 1**	**+**	**+**	-	**+**	**+**	**+**	-	**+**	-	**+**

**Ti-Zr 2**	**+**	**+**	-	**+**	**+**	**+**	-	**+**	-	**+**

**Ti-Zr 3**	**+**	**+**	-	**+**	**+**	**+**	-	**+**	-	**+**

**ZrO**_**2 **_**1**	**+**	**+**	-	**+**	**+**	**+**	-	**+**	-	**+**

**ZrO**_**2 **_**2**	**+**	**+**	-	**+**	**+**	**+**	-	**+**	-	**+**

**ZrO**_**2 **_**3**	**+**	**+**	-	**+**	**+**	**+**	-	**+**	-	**+**

optical interference. - no interference detected; (+) slight interference detected; + interference detected

We therefore modified the DCF and MTT assay protocols and applied the NM dispersions either for 1 h (oxidative stress) or for 24 h (metabolic activity) but removed them from the cell monolayer prior to the incubation with DCF or MTT. These assay adaptations allowed the detection of cytotoxicity endpoints but eliminated NM interference with the measurement of DCF and reduced MTT when particle concentrations were limited to a maximum of 10 μg cm^-2 ^(Table 2). Nevertheless, at 10 μg cm^-2 ^Carbon Black still strongly interfered with light absorption of MTT (Table 2, see Additional file 1: Figure S2 for details). Recently, Carbon Black has also been suggested to specifically adsorb MTT-formazan thereby distorting the assay outcome [10]. We therefore omitted MTT assays for the detection of Carbon Black cytotoxicity. For assessing the influence of Carbon Black on intracellular ROS formation, we performed DCF assays also with 10 μg cm^-2 ^dispersions although a slight reduction of DCF fluorescence due to particle interference was observed at this NM concentration (Additional file 1: Figure S2). Light absorption of INT determined with mixtures of reduced and oxidized INT (for details see Experimental section) was altered by all NM dispersions when these were applied at concentrations of 50 μg cm^-2 ^and above, but not at concentrations of 10 μg cm^-2 ^(Table 2) and below (data not shown). To avoid particle interference during the toxicity screening, we thus limited the applied particle concentration to a maximum of 10 μg cm^-2 ^corresponding to 32 μg ml^-1^.

The catalytic activity of the NM dispersions towards the substrates H_2_DCF-DA, oxidized MTT, and oxidized INT was measured in empty 96-well plate wells pre-incubated with NM dispersions at particle concentrations of 0.01, 0.1, 1 and 10 μg cm^-2^. After 1 h incubation with the substrate H_2_DCF-DA, DCF fluorescence was not detectable with any of the particles used (Table S1). However, 4 h after the addition of H_2_DCF-DA, Carbon Black had produced significant amounts of fluorescent DCF (Table S1). We therefore limited the incubation with H_2_DCF-DA in the assay protocol to 1 h and directly measured DCF production when assessing particle-induced ROS formation. Since none of the particle dispersions catalyzed the reduction of MTT and INT within 24 h (Table S1), no further adaptations of the MTT and LDH assay was required.

Finally, we analyzed the effect of nanomaterial dispersions (from 0.01 to 10 μg cm^-2^) on LDH activity in cell-free wells and did not detect any influence on LDH activity (data not shown).

In summary, our results demonstrate that classical cytotoxicity assays such as MTT, LDH and DCF are not suitable for testing engineered nanomaterials without prior evaluation and adaptation. Due to their intrinsic optical activity, particle concentrations of 50 μg cm^-2 ^heavily influenced the measurement and led either to false positive or to false negative results dependent on the assay used. We therefore introduced a washing step to reduce the amount of particles present during the measurement and limited the particle concentration to 10 μg cm^-2 ^since particles cannot be entirely removed by washing. These results define two essential requirements for NM *in vitro *testing: first, each particle has to be analyzed for assay interference. Second, NM concentrations higher than 10 μg cm^-2 ^potentially lead to false positive/negative results in the selected assays.

#### Particle concentrations

A major challenge in NM *in vitro *testing is to consider the relevance of utilized particle concentrations. *In vivo *doses cannot be converted directly to *in vitro *concentrations. We therefore used *in vivo *studies on test animals to estimate our *in vitro *concentrations in comparison to reported lung burdens. In animal inhalation studies, a so-called overload dose results into exceedance of the macrophage clearance capacity [22]. For TiO_2 _NMs, overload effects in rats were reported at 10 mg m^-3 ^[23,24]. Typical maximum lung burdens were ~ 10 mg g^-1 ^lung (reached after 6 h exposure, [23,24]). With a typical rat lung weighing ~ 1 g [1,25] and the total representative rat lung surface area at 100% lung capacity having been estimated to be ~ 3000 cm^2 ^up to ~ 3500 cm^2 ^[26,27], this lung burden amounts to approximately 3 μg cm^-2 ^NMs per cm^2 ^lung surface. Hence, the applied *in vitro *concentrations from 0.1 μg cm^-2 ^to 10 μg cm^-2 ^cover the range up to overload *in vivo *doses, and might provide valuable information regarding the dose dependency of particle toxicity.

### Selection of cell lines

Previous studies suggested that the cytotoxic response upon exposure to NMs is different in individual cell lines [12,16,28]. Primary cells used were mostly alveolar macrophages which were in some cases more in other less sensitive than immortalized cells [16]. However, most of the studies conducted so far were based on one or two cell lines only. To obtain a more comprehensive view of the biological impact of NMs, we selected ten different and highly characterized commercially available standard cell lines representing varying routes of exposure and well-known toxicity models. Primary cells were not included in the study because we intended to analyze cell models, which are available in constant standardized quality and high quantity. In addition, co-culture models were not used since here we focused on cell viability-related assays instead of assessing NM-induced proinflammatory cell responses.

Four of the selected cell lines were of human origin and derived from organs directly exposed to NMs upon inhalation (lung: A549, CaLu3), dermal application (skin: HaCaT), or ingestion (colon: CaCo2). Six cell lines were of animal origin including lung epithelial cells (RLE-6TN), fibroblasts (NIH-3T3), macrophages (RAW264.7) as well as three different epithelial cell lines (MDCK, MDCK II, NRK52E) representing kidney as an important secondary target organ. For the NM toxicity screening, all cell types were seeded 24 h prior to the exposure to NMs. To ensure constant cell culture quality, metabolic activity and cell cycle state were analyzed regularly.

Although the set of cell lines used was a representative selection of common *in vitro *toxicity models, we cannot exclude that other cell lines may exhibit different responses to NM exposure and may be more effective in the detection of biological effects of NMs.

### *In vitro *toxicity screening

**Figure 1:**
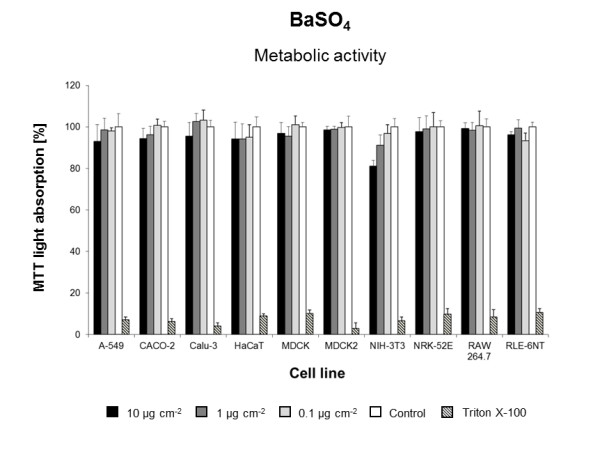
**BaSO**_**4**_**-induced reduction of cell viability**. Metabolic activity in ten cell lines exposed to different concentrations of BaSO_4 _in DMEM/10% FBS or pure DMEM/10% FBS (Control). Data are expressed as% of control mean ± SD of three independent experiments with four replications each. * significantly different from control for p = 0.05.

**Figure 2:**
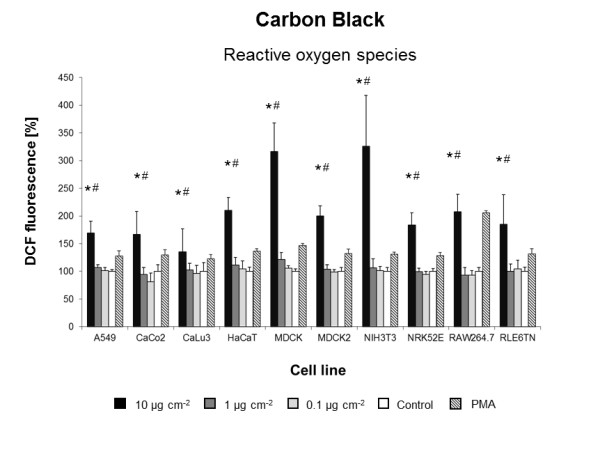
**Carbon Black-induced ROS formation**. DCF fluorescence in ten cell lines exposed to different concentrations of Carbon Black in DMEM/10% FBS or pure DMEM/10% FBS (Control). Data are expressed as% of control mean ± SD of three independent experiments with seven replications each. * significantly different from control for p = 0.05; # significantly different from right neighbor for p = 0.05.

**Figure 3:**
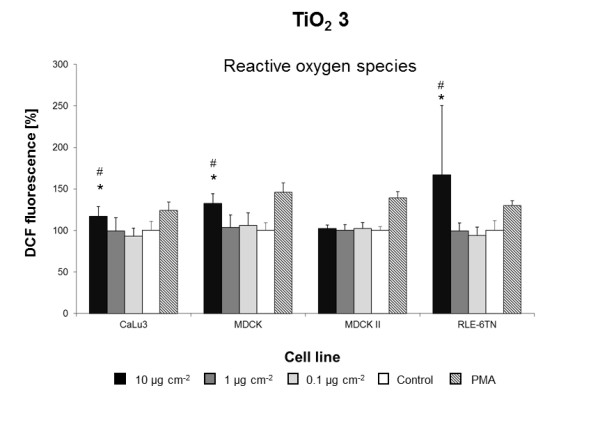
**TiO**_**2 **_**3-induced ROS formation**. Mean DCF fluorescence in ten cell lines exposed to different concentrations of TiO_2 _3 in DMEM/10% FBS or pure DMEM/10% FBS (Control). Data are expressed as% of control mean ± SD of three independent experiments with seven replications each. * significantly different from control for p = 0.05; # significantly different from right neighbor for p = 0.05.

**Figure 4:**
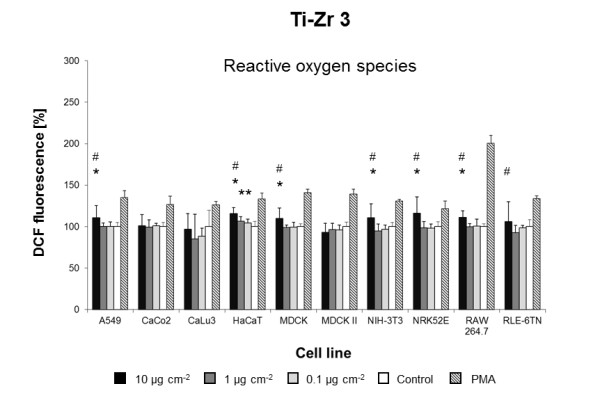
**Ti-Zr 3-induced ROS formation**. Mean DCF fluorescence in ten cell lines exposed to different concentrations of Ti-Zr 3 in DMEM/10% FBS or pure DMEM/10% FBS (Control). Data are expressed as% of control mean ± SD of three independent experiments with seven replications each.* significantly different from control for p = 0.05; # significantly different from right neighbor for p = 0.05.

**Figure 5:**
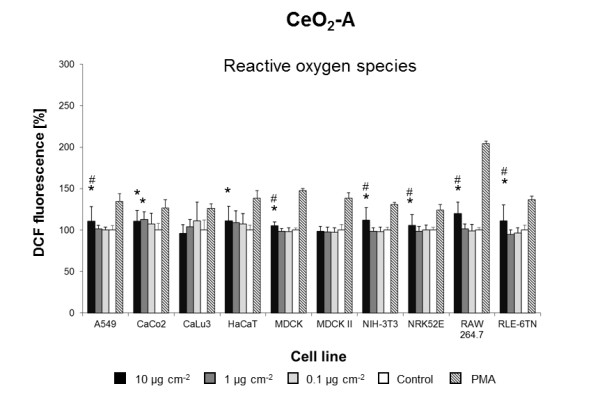
**CeO**_**2 **_**A-induced ROS formation**. Mean DCF fluorescence in ten cell lines exposed to different concentrations of CeO_2 _A in DMEM/10% FBS or pure DMEM/10% FBS (Control). Data are expressed as% of control mean ± SD of three independent experiments with seven replications each. * significantly different from control for p = 0.05; # significantly different from right neighbor for p = 0.05.

**Figure 6:**
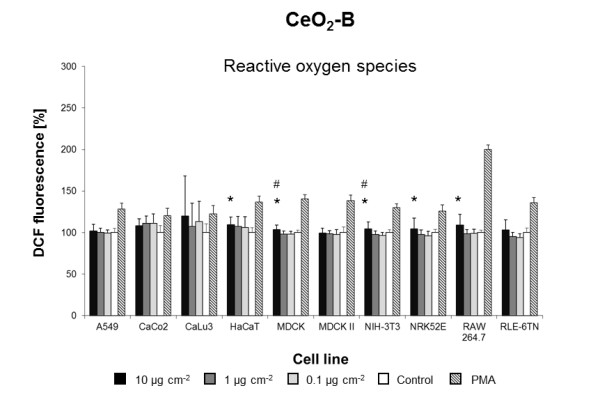
**CeO**_**2 **_**B-induced ROS formation**. Mean DCF fluorescence in ten cell lines exposed to different concentrations of CeO_2 _B in DMEM/10% FBS or pure DMEM/10% FBS (Control). Data are expressed as% of control mean ± SD of three independent experiments with seven replications each. * significantly different from control for p = 0.05; # significantly different from right neighbor for p = 0.05.

**Figure 7:**
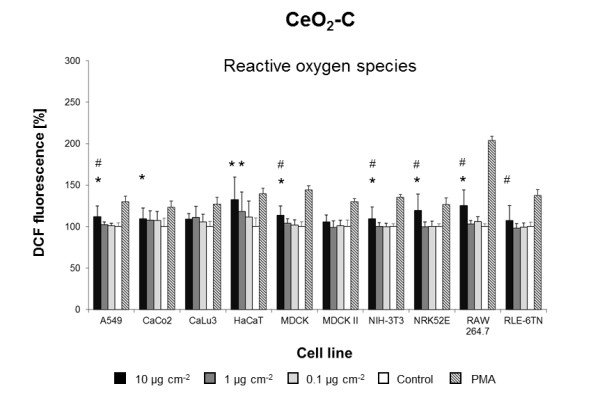
**CeO**_**2 **_**C-induced ROS formation**. Mean DCF fluorescence in ten cell lines exposed to different concentrations of CeO_2 _C in DMEM/10% FBS or pure DMEM/10% FBS (Control). Data are expressed as% of control mean ± SD of three independent experiments with seven replications each. * significantly different from control for p = 0.05; # significantly different from right neighbor for p = 0.05.

Using the three previously modified and standardized *in vitro *assays, we screened the biological effects of 23 engineered NMs in ten different cell lines. Overall, seven NMs induced cytotoxic responses in one or more cell lines tested (Figures 1,2,3,4,5,6,7) whereas all other NMs used for the *in vitro *screening did not show any significant effect at concentrations from 0.1 to 10 μg cm^-2 ^(Additional file 1: Figure S3). Intracellular ROS formation was most frequently observed after exposure to NMs. Significant increases in cell death were not detected with any of the particle types utilized (Additional file 1: Figure S3) and reduced metabolic activity was only displayed by a single cell line NIH3T3) and only when exposed to dispersions of BaSO_4 _NMs (Figure 1, Table 3). *In vivo*, BaSO_4 _inhalation or instillation may act as pulmonary irritant, cause benign and reversible conditions of the lung, or renal and cardiovascular effects [29].

**Table 3:** Summary of the *in vitro *toxicity screening results of the 23 types of NMs screened

	Oxidative stress	Metabolic activity	Cell death
**AlOOH I**	○	○	○

**AlOOH II**	○	○	○

**Al-Ti-Zr 1**	○	○	○

**Al-Ti-Zr 2**	○	○	○

**Al-Ti-Zr 3**	○	○	○

**BaSO**_**4**_	○	**+**	○

**Carbon Black**	**++**	○	○

**CeO**_**2**_	○	○	○

**CeO**_**2 **_**A**	**+**	○	○

**CeO**_**2 **_**B**	**+**	○	○

**CeO**_**2 **_**C**	**+**	○	○

**CeO**_**2 **_**D**	○	○	○

**SrCO**_**3 **_**1**	○	○	○

**SrCO**_**3 **_**2**	○	○	○

**TiO**_**2 **_**1**	○	○	○

**TiO**_**2 **_**2**	○	○	○

**TiO**_**2 **_**3**	**+**	○	○

**Ti-Zr 1**	○	○	○

**Ti-Zr 2**	○	○	○

**Ti-Zr 3**	**+**	○	○

**ZrO**_**2 **_**1**	○	○	○

**ZrO**_**2 **_**2**	○	○	○

**ZrO**_**2 **_**3**	○	○	○

++ reaction observed in all cell lines, + reaction observed in one or more cell line(s), ○ no reaction observed.

#### Nanomaterials induced oxidative stress

Six of the 23 NMs used for the *in vitro *screening induced an increase in ROS formation in five or more cell lines tested (Figure 2,3,4,5,6,7). **Carbon Black**, which we used as reference material, was the only particle that significantly increased the intracellular production of ROS in all ten cell lines when applied at a concentration of 10 μg cm^-2 ^(Figure 2).

The ROS generating potential of Carbon Black has been reported in various studies [30-32]. Comparable to our data, L'Azou et al. recently measured intracellular ROS formation using the same *in vitro *assay (DCF) and Carbon Black NMs from the same supplier with similar primary particle size and surface area (13 nm, 350 g m^-2^). Well in line with our findings, they observed a significant increase in oxidative stress in two renal cell lines (IP I 5 and LLC-PK) after 6 h exposure to 5 μg cm^-2 ^[12]. The carbon Black concentration of 1 μg cm^-2 ^represented the no effect level in our study.

Except for MDCKII, **TiO**_**2 **_**3 **NMs induced a concentration-dependent increase in ROS in all cell lines tested. Effects of TiO_2 _3 on MDCK, MDCKII, CaLu3 and RLE-6TN are displayed in Figure 3, whereas TiO_2 _3-induced ROS formation in A549, CaCo2, HaCaT, NRK-52E and RAW264.7 has been published earlier (see Landsiedel et al. 2010[21], Figure 5B, TiO_2 _B). TiO_2 _1 and 2 NMs did not trigger ROS formation or any other cytotoxic response (see Additional file 1: Figure S3 and Landsiedel et al. 2010 [21], Figure 5A). TiO_2 _1 and 2 NMs display higher anatase content than TiO_2 _3 NMs despite anatase TiO_2 _NMs have been reported to be more biologically active than rutile TiO_2 _NMs in terms of cytotoxicity (for review see [13]). TiO_2 _1 and 2 NMs also differ slightly from TiO_2 _3 NMs regarding size, surface area and crystal morphology. Their primary particle size is below 20 nm, a critical size that can lead to an enhancement of interfacial reactivity thereby modifying biological effects [33]. Hence, these physicochemical properties would enhance the potential biological effects of TiO_2 _1 and 2 NMs compared to TiO_2 _3 NMs. It is therefore likely that the organic surface modification present in TiO_2 _1 and 2 NMs, but not in TiO_2 _3 NMs strongly impairs their ROS generating potential. Recently, Pan et al. reported that intracellular ROS formation was reduced to the control levels when rutile TiO_2 _particles were coated with a dense polymer brush [34]. These findings and our data suggest a fundamental role of particle coatings in modifying cytotoxic effects of TiO_2 _NMs.

The **mixed oxide Ti-Zr 3 **NMs induced an increase in ROS in seven of ten cell lines when concentrations of 10 μg cm^-2 ^were used (Figure 4). No impact on ROS formation was observed for Ti-Zr 1 and 2 NMs in any of the ten cell lines tested (Additional file 1: Figure S3). The Ti-Zr mixed oxide NMs 1 to 3 were of similar primary particle sizes and surface areas, but consisted of different ratios of ZrO_2 _to TiO_2 _(Table 1). Of the three mixed oxide NMs tested, Ti-Zr 3 NMs contained the largest amount of TiO_2 _(90%). This strongly suggests that the TiO_2 _fraction is driving cytotoxicity of Ti-Zr 3 NMs. Recently, Bar-Ilan et al. demonstrated that gold and silver NMs of similar size and surface area displayed significantly different toxic effects suggesting that chemical composition is as important as a specific NM surface area in inducing NM toxicity [35]. Furthermore, Limbach et al. have shown that the chemical composition is one of the key attributes of engineered NMs determining the formation of ROS in exposed cells [36]. We therefore assume that the differences in chemical composition of the mixed oxide Ti-Zr NMs are mostly responsible for the observed differences in ROS induction.

Three of the five **CeO**_**2 **_NMs (**CeO**_**2 **_**A-C) **induced intracellular ROS formation in five to eight cell lines tested (Figures 5,6,7). CeO_2_-A to D NMs were from the same supplier and generated by varying parameters of the same production process (Table 1). At a concentration of 10 μg cm^-2^, CeO_2_-A and CeO_2_-C NM dispersions significantly induced ROS in eight of the ten cell lines (Figures 5 and 7). After exposure to CeO_2_-B NM dispersions, an increase in ROS formation was observed in five cell lines (Figure 6). Most strikingly, CeO_2_-D NMs did not evoke a significant increase of ROS in any of the ten cell lines tested (Additional file 1: Figure S3). However, CeO_2_-D particles are almost identical to CeO_2_-A NMs except slight differences in the surface chemistry due to variations in gassing with carbon dioxide during the production process (Table 1). Importantly, these differences in surface chemistry result in a lower ζ-potential and pH in H_2_O (Table 1). Prior to the cell exposure, however, all NMs were dispersed in cell culture medium and displayed the same ζ-potential and pH (Table 1). Nevertheless, differences in surface chemistry may lead to a different adsorption of proteins present in the cell culture medium. Studies on CeO_2 _NMs with different ζ-potentials revealed that electrostatic interactions seem to be the main driving force in protein adsorption and cellular uptake [37]. Protein adsorption has been suggested to be as influential in driving toxicity as the inherent physicochemical properties of NMs [38]. Moreover, Berg et al. recently demonstrated that changes in the ζ-potential of metal oxide NMs are associated with changes in cell viability [39]. Our results strongly suggest that slight differences in surface chemistry play an important role in triggering CeO_2 _NM effects in cells.

The comparative study of thoroughly characterized NMs allowed us to identify NM material properties that contribute to NM cytotoxicity. From the 23 engineered NMs tested, we found organic surface modification (TiO_2 _NMs), chemical composition (Ti-Zr mixed oxide NMs), and surface chemistry (CeO_2 _A-D NMs) to be associated with biological activity.

#### Sensitivity of individual cell lines

**Figure 8:**
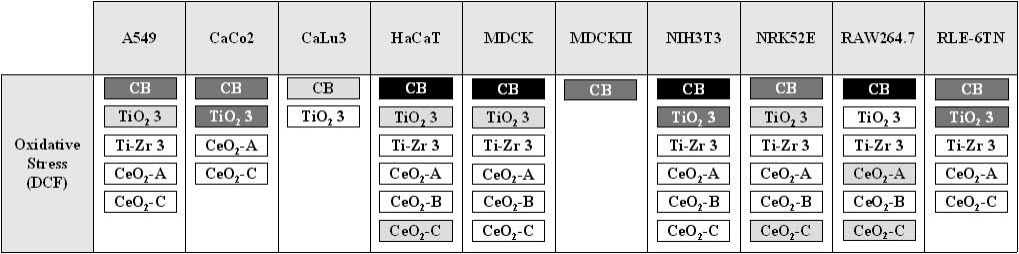
**Summary of the cell-line specific oxidative responses after exposure to different NMs**. Mean DCF fluorescence values significantly different from control are displayed as follows: black: >200% of control; dark grey: 150-200% of control; light grey: 120-150% of control; white: >100-120% of control.

The responsiveness of the cell lines used differed when exposed to NM under identical culture and exposure conditions. This phenomenon is here referred to as sensitivity of cell lines. The tested cell lines showed individual patterns of ROS formation upon exposure to the different NM dispersions (Figures 2,3,4,5,6,7). Of the three kidney cell lines used in this study, MDCK and NRK52E displayed an increase in ROS formation after exposure to 10 μg cm^-2 ^of six different NMs whereas in MDCK II, ROS formation was induced only by Carbon Black (Figure 8). MDCK cells have been shown to contain two cell strains, MDCK I and MDCK II, with different functional and structural characteristics [40].

We also found a different sensitivity towards NMs in the three lung derived epithelial cell lines that we used in this study. Overall, the cell line RLE-6TN exhibited the strongest increase in ROS (Figures 2,3,4,5,6,7). However, most of the studies reported so far have been performed with A549, which display features of type I and type II lung alveolar epithelial cells [41]. We observed that ROS formation in these cells was slightly lower than that in RLE-6TN cells after exposure to four of the six NMs with ROS inducing potential (Figures 2,3,4,5,6,7). The murine peritoneal macrophage cell line RAW264.7 displays similarities with alveolar macrophages [42] and has been widely used in studies investigating the pulmonary toxic potential of particulate matter [e.g. [43,44] and NMs [e.g. [45,46]. Similar to previous reports, we observed a high degree of ROS formation in these cells after exposure to different NMs. Except for TiO_2 _3 NMs, sensitivity of RAW264.7 towards the NMs that induced ROS formation was higher than that displayed by A549 or RLE-6TN (Figure 8). The lowest effects were observed in CaLu3 cells, which derived from human bronchial epithelium [47]. In these cells, we only found an oxidative stress response when we exposed the cells to dispersions of 10 μg cm^-2 ^Carbon Black or TiO_2 _3 NMs (Figure 8). Similarly, individual reaction patterns upon exposure to different NMs have been observed in a bronchial and an alveolar epithelial cell line (BEAS-2B, A549) [16]. These findings and our data suggest that even when assessing NM toxicity to cells originating from the same organ, the outcome highly depends on the cell type used.

To evaluate the impact of NM exposure on gut-derived cells we used CaCo2 cells that have been established from a human colon carcinoma and display differentiation features characteristic for mature intestinal cells [48]. We found a medium stress response in these cells induced by four of the six NMs inducing ROS production (Figure 8).

HaCaT keratinocytes were derived from normal human skin and have spontaneously immortalized, but have retained the full epidermal differentiation capacity [49]. The mouse fibroblast cell line NIH-3T3 stems from an embryonic culture and is highly contact-inhibited [50]. In this study, both cell lines displayed a high degree of sensitivity towards NMs since ROS formation was observed with all of the six ROS-inducing NMs (Figure 8). Moreover, NIH-3T3 was the only cell line in which we found a decrease in metabolic activity after exposure to dispersions of BaSO_4 _(Figure 1).

Cell culture quality controls showed that the doubling time was similar in all cell lines tested (22 to 24 h). Thus, the observation that cell lines with long doubling times may be more sensitive to NMs [28,51] is not applicable to our study.

Taken together, we found a high degree of cell line-specific responsiveness under the selected culture and exposition conditions suggesting that the use of different cell lines is essential to obtain relevant data. A minimal set of five cell lines, i.e. HaCaT, MDCK, NIH-3T3, NRK52E, and RAW264.7, would have been sufficient in our study to comprehensively characterize the biological impact of the 23 NMs used here (Figure 8).

## Conclusions

Here, we performed a screening of 23 engineered NMs using a matrix of ten different cell lines and three standardized *in vitro *assays measuring different cytotoxicity endpoints. A major outcome of our study is that only seven of 23 NMs tested displayed biological effects at a concentration of 10 μg cm^-2^, which is already beyond exposure concentrations used for *in vivo *studies. At lower concentrations (1 μg cm^-2 ^and below), which better reflect *in vivo *doses, only four NMs induced significant cell responses in single cell lines. ROS formation was by far the most prominent effect observed and, apart from one exception, none of the NMs tested induced cell death or reduced metabolic activity under the experimental conditions used in this study.

From our findings and previous reports it can be concluded that the biological impact of NMs result from the combined effects of a variety of physicochemical properties including chemical composition, size, surface coating, and surface chemistry. This impairs a precise prediction of NM toxicity and emphasizes the need for a thorough physicochemical characterization and toxicity testing of each type of NM.

Finally, our results demonstrate that sensitivity towards nanomaterial exposure is not only cell-type-specific, but also depends on the particle type used as well as on the toxicity endpoint measured. We therefore suggest an *in vitro *testing strategy based on a matrix that includes both a series of suitable cell lines and a set of standardized cytotoxicity assays to obtain a more comprehensive view of NM biological activity. Depending on the pathomechanisms of interest, other endpoints like inflammatory response or genotoxicity and different sets of cell lines or co-culture models may be applied. A highly sensitive cytotoxicity test system has the potential to reduce the amount of animal studies by preselecting cytotoxic NMs, which require further hazard assessment.

## Methods

### Chemicals

If not otherwise stated, all chemicals were obtained from Sigma-Aldrich Chemie GmbH. In particular, lactate dehydrogenase (LDH), 3-(4,5-dimethylthiazol-2-yl)-2,5-diphenyl-tetrazoliumbromide (MTT), 2-(4-iodophenyl)-3-(4-nitrophenyl)-5-phenyl tetrazolium chloride (INT), phorbol 12-myristate 13-acetate (PMA), and β-nicotinamide adenine dinucleotide hydrate (NAD) were obtained from Sigma-Aldrich Chemie GmbH. 2',7'-dichlorodihydrofluorescein diacetate (H_2_DCF-DA) was obtained from Invitrogen GmbH. Sodium bicarbonate and sodium pyruvate were from Lonza GmbH. Phenol red free DMEM, DMEM/Ham's F12, RPMI 1640, and FBS Gold were obtained from PAA Laboratories GmbH. Phenol red free MEM was obtained from Biochrome AG and phenazinmethosulphate (PMS) and lactic acid were from Fluka/Sigma-Aldrich Chemie GmbH. Engineered NMs were part of the NanoCare portfolio of materials [18].

### Cell lines and culture conditions

The cell lines used for this study were obtained from ATCC (American Type Culture Collection; A549, CaCo-2, CaLu3, MDCK (NBL-2), RAW264.7, and RLE-6TN), CLS Cell Line Service (HaCaT), DSMZ (German Resource Centre for Biological Material; NIH-3T3, NRK-52E), and ECACC (European Collection of Cell Cultures; MDCK II). The authenticity of the human cell lines was determined by the DSMZ via DNA fingerprinting. The cell lines were cultured in the following standard media: DMEM with 10% FBS Gold and 4 mM L-glutamine (A549, HaCaT, NRK-52E, RAW264.7), DMEM with 20% FBS Gold and 4 mM L-glutamine (CaCo-2), DMEM with 10% FBS Gold (NIH-3T3), RPMI with 10% FBS Gold, 2 mM L-glutamine and 1 mM sodium pyruvate (CaLu3, MDCK (NBL-2), RLE-6TN) or MEM with 5% FBS Gold and 2 mM L-glutamine (MDCK II). Cultured cells were propagated at 37°C and 5% CO_2_. Cells were grown to confluence within three to four days and then transferred to new culture plates. NIH-3T3 fibroblasts were maintained subconfluent at all times. Cells were used for up to passage 20 and regularly tested by PCR for mycoplasma infection.

The cell lines were all found to reach confluence after 24 h of cultivation when seeded at 3 × 10^4 ^(RAW264.7: 6 × 10^4^) cells per well in a 96-well plate (Additional file 1: Figure S4). Effectual cell adhesion and spreading was controlled via fluorescence and phase contrast imaging with cells seeded onto coverslips (according to cell numbers seeded in 96-well plates) that were were rinsed twice with Dulbecco's phosphate buffered saline (PBS) and fixated chemically (45 min at room temperature) using 1% neutral buffered formalin after 24 h of cultivation. Filamentous actin (F-actin) was labelled with Alexa Fluor 488 phalloidin (Invitrogen, Darmstadt, Germany) and nuclei were stained with 4',6-Diamidino-2-phenylindole dihydrochloride (DAPI, Sigma-Aldrich Chemie GmbH, Steinheim, Germany).

Images were captured using an inverse fluorescence microscope Olympus IX81 in combination with a 40x/0.60 microscope objective (Olympus LUC Plan FLN PH2). Phase contrast images (exposure time 20 ms) and fluorescence images (FITC filter, exposure time 1500 ms and DAPI filter, exposure time 50 ms, respectively) were captured with the CCD sensor F-View II (1376 × 1032 pixels) and processed using the cell^p software (all Olympus Deutschland GmbH, Hamburg, Germany, Additional file 1: Figure S4).

Adhesion and confluence of cell lines of epithelial origin was further confirmed by measuring the total impedance of cell layers non-invasively using the Electric Cell-Substrate Impedance Sensing (ECIS) system (ECIS™ Model 1600, Applied Biophysics, Troy, NY, USA). To this end, 66000 cells per well (corresponding to 3 × 10^4 ^cells per well area in a 96-well plate) were seeded in 8W10E impedance arrays (ECIS™ Culture ware) and monitored for 50 h at 45 kHz. Except for CaLu3 cells, all cell lines reached a stable impedance level within 25 to 48 h (data not shown, NM-induced oxidative cell stress was measured after 25 h, metabolic activity 48 h and cell viability at 48 h). A stable level of impedance indicates a confluent layer without drastic morphological alterations.

#### Cell culture quality control

In order to monitor the development of metabolic activity and cell number in the untreated cultures in the exposure medium, all cell lines were seeded at 3 × 10^4 ^(RAW264.7: 6 × 10^4^) cells per well in 96-well plates in the cell culture media as specified above. After 24 h, the specific media were exchanged for the exposure medium DMEM with 10% FBS Gold and 4 mM L-glutamine and samples were taken immediately, after 48 h, 72 h, and 96 h. The metabolic activity was determined in three wells per cell line by the MTT assay (see below), while the number of cells was determined by a CASY^® ^Model TT (Innovatis AG, Reutlingen, Germany). The ratio of metabolic activity and cell number remained constant in CaCo2, MDCK II, and RLE-6TN, while it dropped slightly after media exchange in the other cell lines (data not shown). The exposure medium was identical to the cell line specific medium for A549, HaCaT, NRK-52E and RAW264.7. The cell cycle state of each cell line was monitored prior to each experiment. An aliquot of the cells seeded for a screening experiment was fixed with 70% ethanol at -20°C over night and stained with 20 μg ml^-1 ^propidium iodide at 37°C for 15 min. Cells were washed with ice cold 1× PBS prior and after fixation and staining. Propidium iodide stained cells were kept on ice in the dark and analyzed immediately by flow cytometry using a COULTER^® ^EPICS^® ^XL™ and XL-MCL™ Flow Cytometer (Beckmann Coulter, N. Harbor Boulevard, USA). The signal intensity of the fluorescing propidium iodide stained cells was plotted against the width of the fluorescent cells. Signals of aggregated cells were excluded from further analysis according to the protocol for cell cycle analysis by propidium iodide staining provided by Beckmann Coulter. The number of the remaining cell signals was plotted against the cell signal intensity resulting into a G1-phase peak, a low S-phase plateau, and a G2-phase peak. The percentage of the cell cycle phases was assessed with WinMDI 2.9 (^© ^Joseph Trotter).

#### Characterization of nanomaterials

Primary particle size, crystallinity, impurities, surface chemistry, and ζ-potential were determined by transmission electron microscopy (TEM), X-ray diffraction (XRD), X-ray photoelectron spectroscopy (XPS), and dynamic light scattering (DLS), respectively, as described previously [18,20,21].

#### Determination of NM solubility

The solubility of NMs was determined via ICP-OES (ISO 11885) by Wessling Holding GmbH & Co. KG, Altenberge, Germany. In particular, NMs were diluted to 32 μg ml^-1 ^in DMEM/10% FBS from stock dispersions and continuously stirred as described previously [20]. Samples were taken 24 h after dilution. All samples were immediately centrifuged for 3 h at 4°C and 20,000 *g*. Detection limits were Ce 5 μg l^-1^, Ti 1 μg l^-1^, Al 3 μg l^-1^, Zr 1 μg l^-1^, Ba 1 μg l^-1^, and Sr 0.1 μg l^-1^.

### Sterilization and dispersion of nanomaterials in cell culture medium

NMs were sterilized and dispersed according to a standardized protocol as described previously [20]. In particular, NM aliquots of 19.2 mg were transferred into sterile snap-lid glasses together with a magnetic stir bar and exposed to 30 Gy gamma irradiation. The dispersions were stirred at 900 rpm for 1 h at room temperature in 6 ml of DMEM/10% FBS. Dilutions of this stock dispersion were prepared immediately and stirred for 24 h at 900 rpm at room temperature.

### Quantification of endotoxin

The concentration of endotoxin in NM dispersions (32 μg ml^-1 ^in DMEM/10% FBS) was quantified with the Limulus Amebocyte Lysate (LAL) Kinetic-QCL^® ^kit (Lonza GmbH, Wuppertal, Germany; 50 - 650 U) as described previously [20]. All NM dispersions were free of endotoxin.

### Detection of reactive oxygen species

To avoid any influence of the possible photocatalytic activity of the NMs on the *in vitro *toxicity tests, all assays were performed in the absence of UV light. Reactive oxygen species (ROS) were detected using the fluorescein derivative H_2_DCF-DA [52]. Cells were seeded in 96-well plates at a density of 3 × 10^4 ^cells per well (RAW264.7: 6 × 10^4 ^cells per well) in a total volume of 100 μl. After 24 h, cells were exposed to NM dispersions (100 μl per well) for 1 h in the absence of the substrate H_2_DCF-DA. Hydrogen peroxide (100 mM) and phorbol 12-myristate 13-acetate (PMA, 4 ng ml^-1^) diluted in stirred DMEM/10% FBS were used as positive controls, stirred DMEM/10% FBS served as negative control. Prior to the addition of the substrate, cells were washed with Krebs-Ringer-Buffer (KRB; pH 7.4, 114 mM NaCl, 3 mM KCl, 1.5 mM K_2_HPO_4 _× 3 H_2_O, 10 mM HEPES, 4 mM D-Glucose, 1.4 mM CaCl_2_, 2.6 mM MgCl_2_). Subsequently, cells were incubated with either 100 μl KRB or 100 μl of assay solution (5 μM H_2_DCF-DA diluted in KRB) for 1 h at 37°C with 5% CO_2_. Finally, cells were washed twice with 100 μl KRB to remove the assay solution and the fluorescence was immediately monitored in a spectrophotometer (excitation 485 nm, emission 520 nm; NOVOstar and FLUOstar, BMG Labtech GmbH, Offenburg, Germany). Each experiment was repeated at least three times with seven replications.

The interference of NMs with the optical detection of DCF fluorescence was assessed by replacing the assay substrate H_2_DCF-DA by defined amounts of fluorescent DCF. Cell monolayers prepared as mentioned above were incubated with 100 μl NM dispersions containing particle concentrations of 0.01, 0.1, 1, 10, 50, or 100 μg cm^-2^, respectively, or stirred cell culture medium (control, 100 μl per well) for 1 h at 37°C. DCF diluted in KRB was added at a final concentration of 100, 50, 10, 1, 0.1, or 0.01 nM DCF, either directly or after the cells had been washed with KRB. Fluorescence was measured immediately as described above to avoid any cell-mediated generation of DCF.

Likewise, the catalytic activity of NM dispersions regarding H_2_DCF-DA oxidation was tested. To this end, empty 96-well plates were incubated with NM dispersions (10, 5, 1, 0.1, or 0.01 μg cm^-2^) or stirred cell culture medium (control) for 1 h at 37°C and washed with KRB before the H_2_DCF-DA assay solution was added. After 1 h incubation at 37°C, the plates were washed twice with KRB, and fluorescence was recorded immediately and after an additional incubation for 3 h at 37°C.

### Quantification of metabolic activity

Metabolic activity was assessed by the reduction of the yellow tetrazolium salt MTT (3-(4,5-dimethylthiazol-2-yl)-2,5-diphenyltetrazoliumbromide) to a purple, insoluble MTT-formazan. Cells were prepared as described above and exposed to NM dispersions for 24 h. Triton X-100 (0.01%) diluted in stirred DMEM/10% FBS served as positive control and was added to untreated cells 30 minutes prior to the end of the incubation. Afterwards, the cells were washed twice with 100 μl PBS and incubated with 2.2 mM oxidized MTT in 110 μl PBS for 2 h. To dissolve the MTT-formazan crystals, 100 μl of 15% SDS/50% DMSO were added. 96-well plates were shaken at 300 rpm over night at 37°C. Light absorption was measured at 550 nm and for reference at 670 nm in a spectrophotometer. Each experiment was repeated three times with four replications.

The interference of NM dispersions with the optical detection of MTT-formazan was analyzed using mixtures of the reaction product (MTT-formazan) and the substrate (oxidized MTT). NM dispersions with particle concentrations of 0.01, 0.1, 1, 10, 50, and 100 μg cm^-2^or stirred cell culture medium (control, 100 μl per well) were added to cell monolayers prepared as described above and incubated for 24 h at 37°C. To follow two different approaches, mixtures of reduced and oxidized MTT were either directly added after NM incubation or the cells were first washed with KRB prior to adding the MTT mixtures (100 μl per well). For the procedure that includes the washing step, 2.4 mM MTT-formazan was diluted to 1.2, 0.8, 0.24, 0.024, and 0.0024 mM with 2.4 mM oxidized MTT to cover the whole spectrum of light absorption. When following the classical protocol, the concentrations of reduced and oxidized MTT were doubled with respect to the dilution. The light absorption at 550 nm was recorded immediately to avoid any cell-based reduction of MTT.

To assess the influence of NM dispersions on the reduction of MTT, empty wells were incubated with NM dispersions (10, 5, 1, 0.1, and 0.01 μg cm^-2^) or stirred cell culture medium (control) and washed as described above. The standard MTT solution (2.4 mM MTT) was added and incubated for 3 h at 37°C followed by the addition of 100 μl SDS/DMSO solution, overnight shaking at 37°C and measurement of the light absorption at 550 nm.

### Measurement of cell death

Cell death was quantified by measurement of lactate dehydrogenase (LDH) activity in cell supernatants. LDH catalyzes the oxidation of lactate in the presence of NAD (β-nicotinamide adenine dinucleotide) yielding pyruvate and NADH + H^+^. The latter in turn reduces INT (2-(4-iodophenyl)-3-(4-nitrophenyl)-5-phenyl tetrazolium chloride) yielding a red water-soluble formazan which was quantified via the measurement of light absorption. Phenazine methosulphate (PMS) served as an electron transfer agent. The protocol was adapted from Korzeniewski and Callewaert [53]. To this end, cell supernatants (50 μl) were mixed with 100 μl assay solution (0.35 g l^-1 ^INT, 0.089 g l^-1 ^PMS, and 0.9 g l^-1 ^NAD in 0.2 M Tris-Base pH 8.2 and 5 g l^-1 ^lactic acid). Cell supernatants from cells treated with Triton X-100 (0.01%) diluted in stirred DMEM/10% FBS served as positive controls. The increase of light absorption at 492 nm was monitored continuously over 1 h at 37°C in a spectrophotometer. Each experiment was repeated three times with four replications.

Interference of NM dispersions with the optical detection of INT reduction was analyzed using different mixtures of reduced and oxidized INT. Cell monolayers prepared as described above were exposed to NM dispersions with particle concentrations of 0.01, 0.1, 1, 10, 50, and 100 μg cm^-2^or stirred cell culture medium (control, 100 μl per well) for 24 h at 37°C. 50 μl of the supernatants were transferred to new 96-well plates and mixtures of reduced INT (1.75, 1.16, 0.35, 0.035, and 0.0035 mM, respectively) with 0.35 mM oxidized INT were added. The light absorption at 492 nm was recorded immediately to avoid any cell-based reduction of INT.

The catalytic activity of NMs was tested in empty wells pretreated with NM dispersions (10, 5, 1, 0.1, and 0.01 μg cm^-2^, respectively) or in stirred cell culture medium (control) as described above. 50 μl of the supernatants were mixed with 50 μl of the LDH assay solution containing 0.35 mM oxidized INT. Light absorption was measured continuously for 1 h at 37°C.

The influence of NM dispersions on LDH activity was determined with defined concentrations of bovine LDH. In particular, NM dispersions or stirred cell culture medium (control) were mixed with LDH to a final concentration of 0.3 U ml^-1 ^and added to empty wells (100 μl per well). After 24 h incubation at 37°C, 50 μl of the supernatants were transferred to new 96-well plates and 50 μl of the LDH assay solution was added. Light absorption at 492 nm was recorded continuously for 1 h at 37°C.

### Calculations and statistical analyses

Data obtained from cells exposed to control medium were equated with 100%. Results are presented as mean values with standard deviations of all experiments. Normal distribution of test results was verified and variance was tested by post hoc one-way ANOVA according to Tukey for p = 0.05.

## Competing interests

The authors declare that they have no competing interests.

## Authors' contributions

AK performed test procedure standardization, endotoxin quantification, *in vitro *toxicity screening by ROS, MTT and LDH assays and manuscript drafting. CD investigated the quality, confluence and adhesion of cultured cells. CD and CR performed impedance measurements and subsequent biophysical data analysis. DH participated in data analysis, study coordination and manuscript drafting. WW, CSI, CG, MV and FH participated in particle characterization by TEM, XRD, XPS, DLS and manuscript drafting. JS conceived the study, participated in its design and coordination and participated in manuscript drafting. All authors read and approved the final manuscript.

## Supplementary Material

Additional file 1 **Screening supporting information revised final.pdf**. Cytotoxicity screening of 23 engineered nanomaterials using a test matrix of 10 cell lines and 3 assays. Contains additional tables and figures as supplementary information Click here for file
