# Supplementary material for: Cytotoxicity screening of 23 engineered nanomaterials using a test matrix of ten cell lines and three different assays
Source: Part Fibre Toxicol. 2011 Feb 23;8:9. doi: 10.1186/1743-8977-8-9 (PMC3059267; doi:10.1186/1743-8977-8-9)
Supplement: Additional file 1 — Screening supporting information revised final.pdf. Cytotoxicity screening of 23 engineered nanomaterials using a test matrix of 10 cell lines and 3 assays. Contains additional tables and figures as supplementary information [file 1743-8977-8-9-S1.PDF]

Additional file 1

Cytotoxicity screening of 23 engineered nanomaterials using a test matrix of ten cell lines and three different assays

Alexandra Kroll[1,7], Christian Dierker[1], Christina Rommel[1], Daniela Hahn[1], Wendel Wohlleben[2], Christian Schulze Isfort[3], Christian Göbbert[4], Matthias Voetz[5], Ferdinand Hardinghaus[6], Jürgen Schnekenburger[1*]

[1]Gastroenterological Molecular Cell Biology, Department of Medicine B, Westfälische Wilhelms-Universität, Münster, Germany

[2]BASF SE, Polymer Physics Research, Ludwigshafen, Germany

[3]Evonik-Degussa GmbH, R & D Aerosil, Hanau, Germany

[4]ItN Nanovation AG, Halberstadt, Germany

[5]Bayer Technology Services, Leverkusen, Germany

[6]Solvay Infra Bad Hönningen GmbH, Bad Hönningen, Germany

[7]present address: Eawag, ETH Domain, Überlandstrasse 133, 8600 Dübendorf, Switzerland

*Corresponding author:

Jürgen Schnekenburger

Gastroenterological Molecular Cell Biology

Department of Medicine B,

Westfälische Wilhelms-Universität

Domagkstraße 3a

48149 Münster, Germany

Tel.: +49 251 8352534

Fax: +49 251 8357938

E-mail: schnekenburger@uni-muenster.de

Particle Characterization

A TiO_2 1

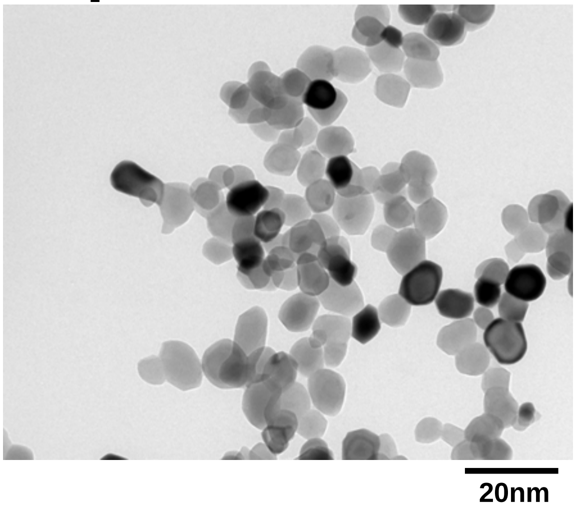

B TiO_2 2
See TiO_2 1

C TiO_2 3

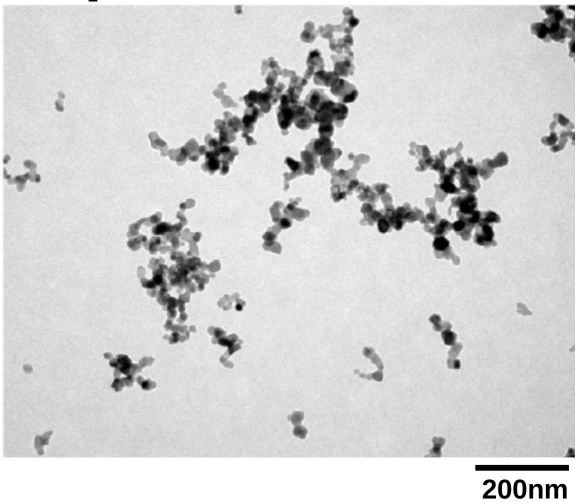

D Carbon Black

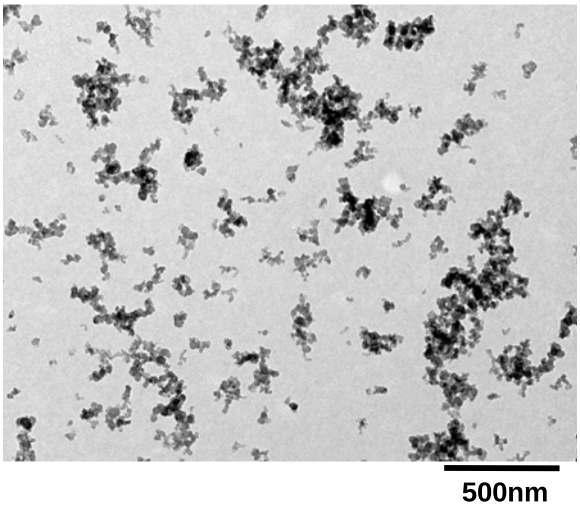

E CeO_2 A

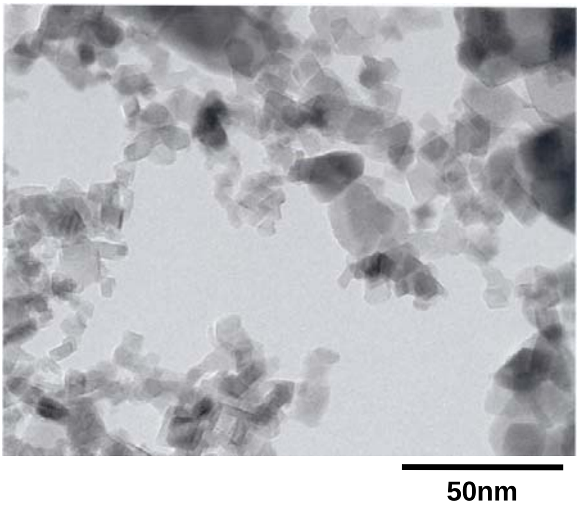

F CeO_2 B

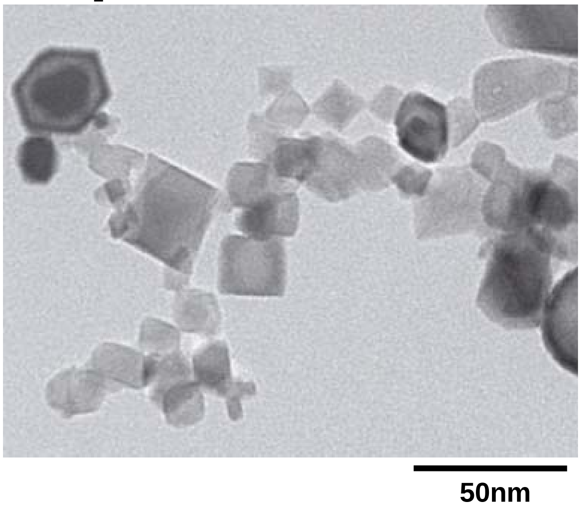

G CeO_2 C

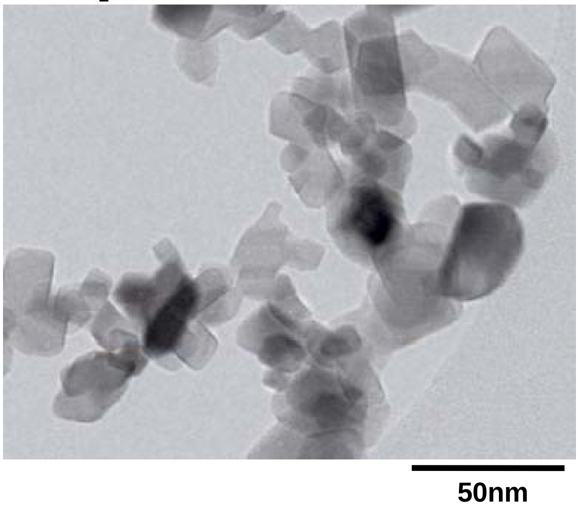

H CeO_2 D

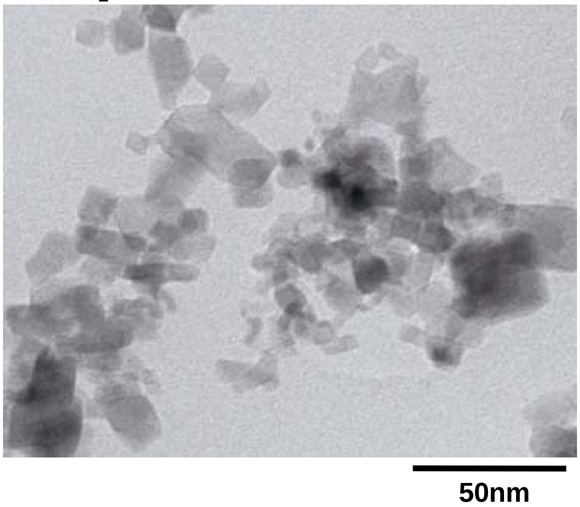

I CeO_2

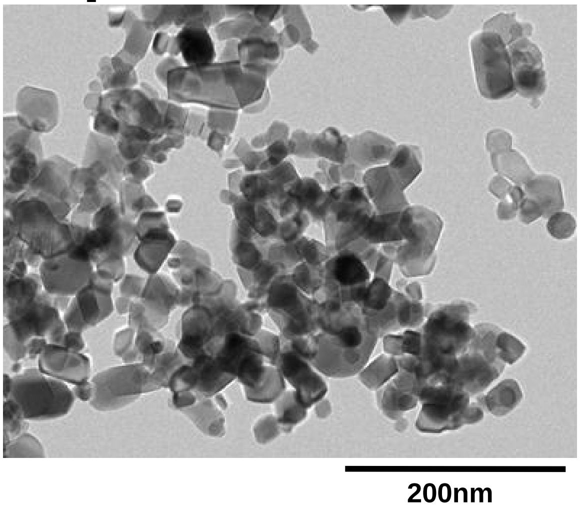

J AlOOH I

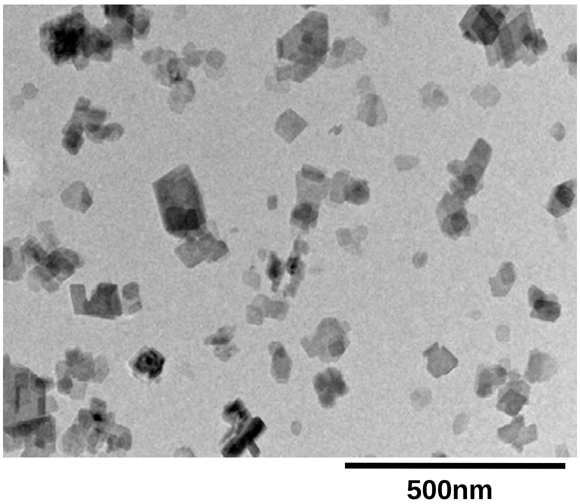

K AlOOH II

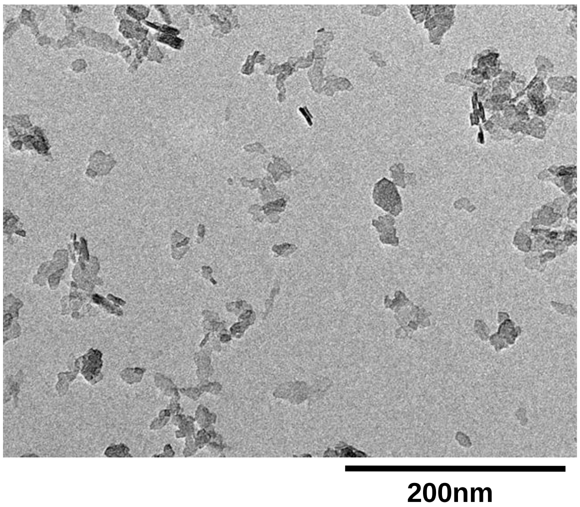

L Ti-Zr 1

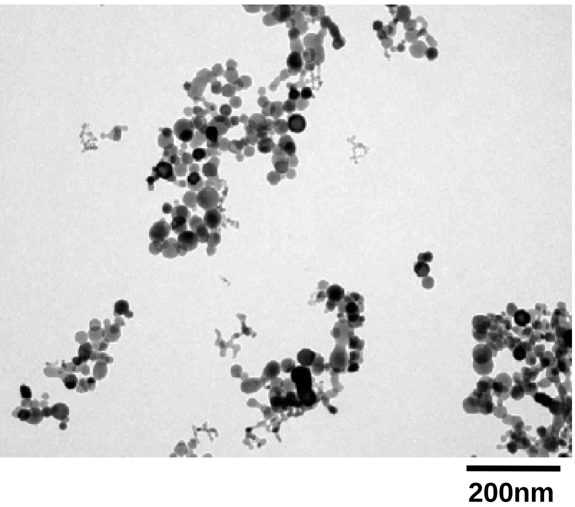

M Ti-Zr 2

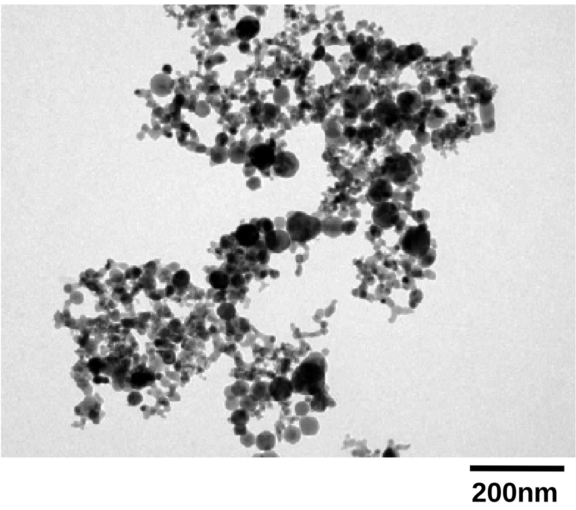

N Ti-Zr 3

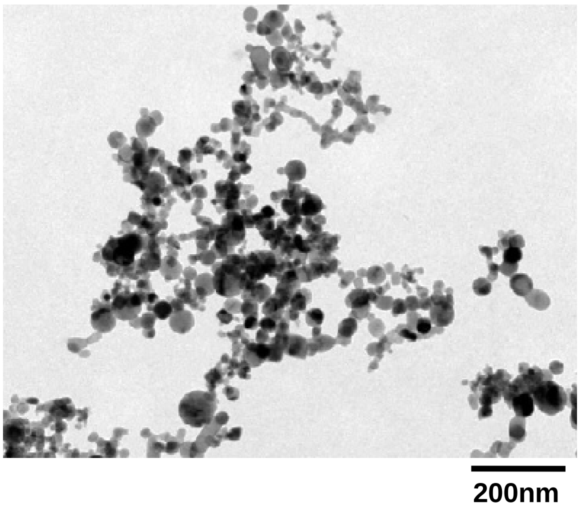

O Al-Ti-Zr 1

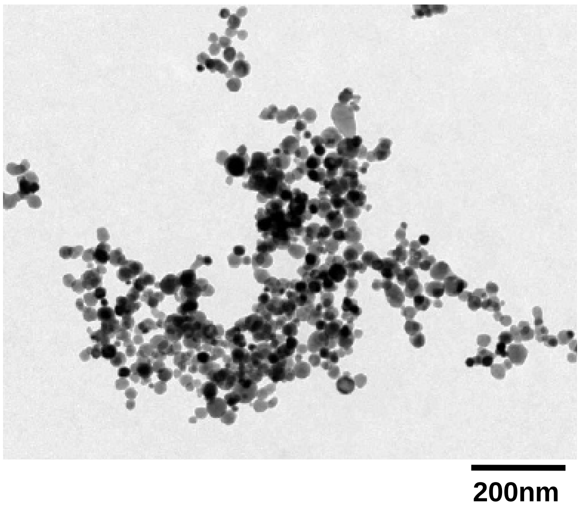

P Al-Ti-Zr 2

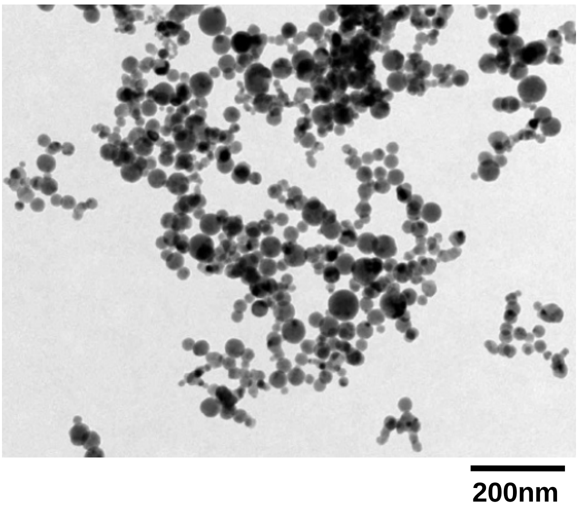

Q Al-Ti-Zr 3

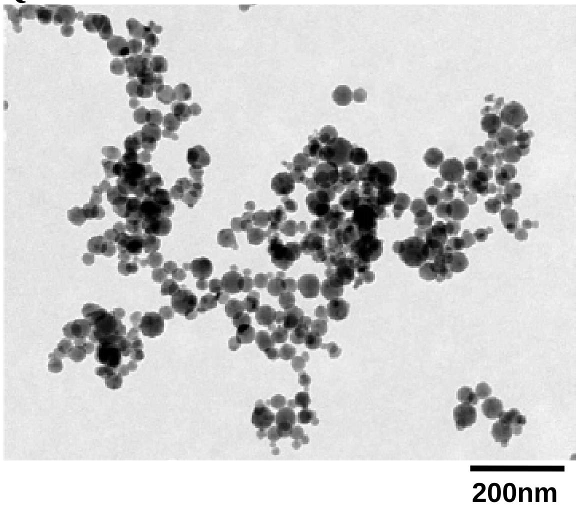

R ZrO_2 1

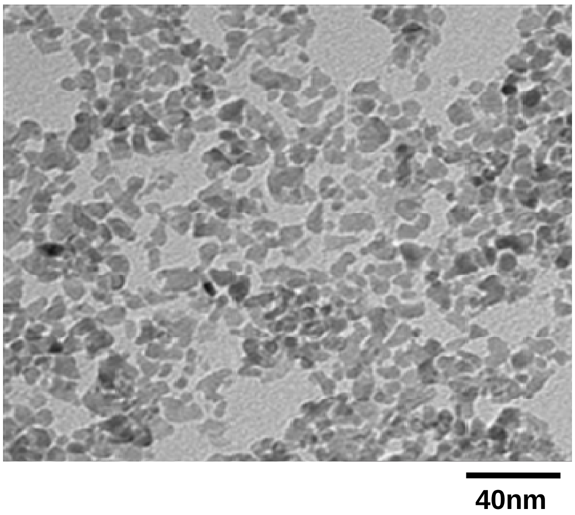

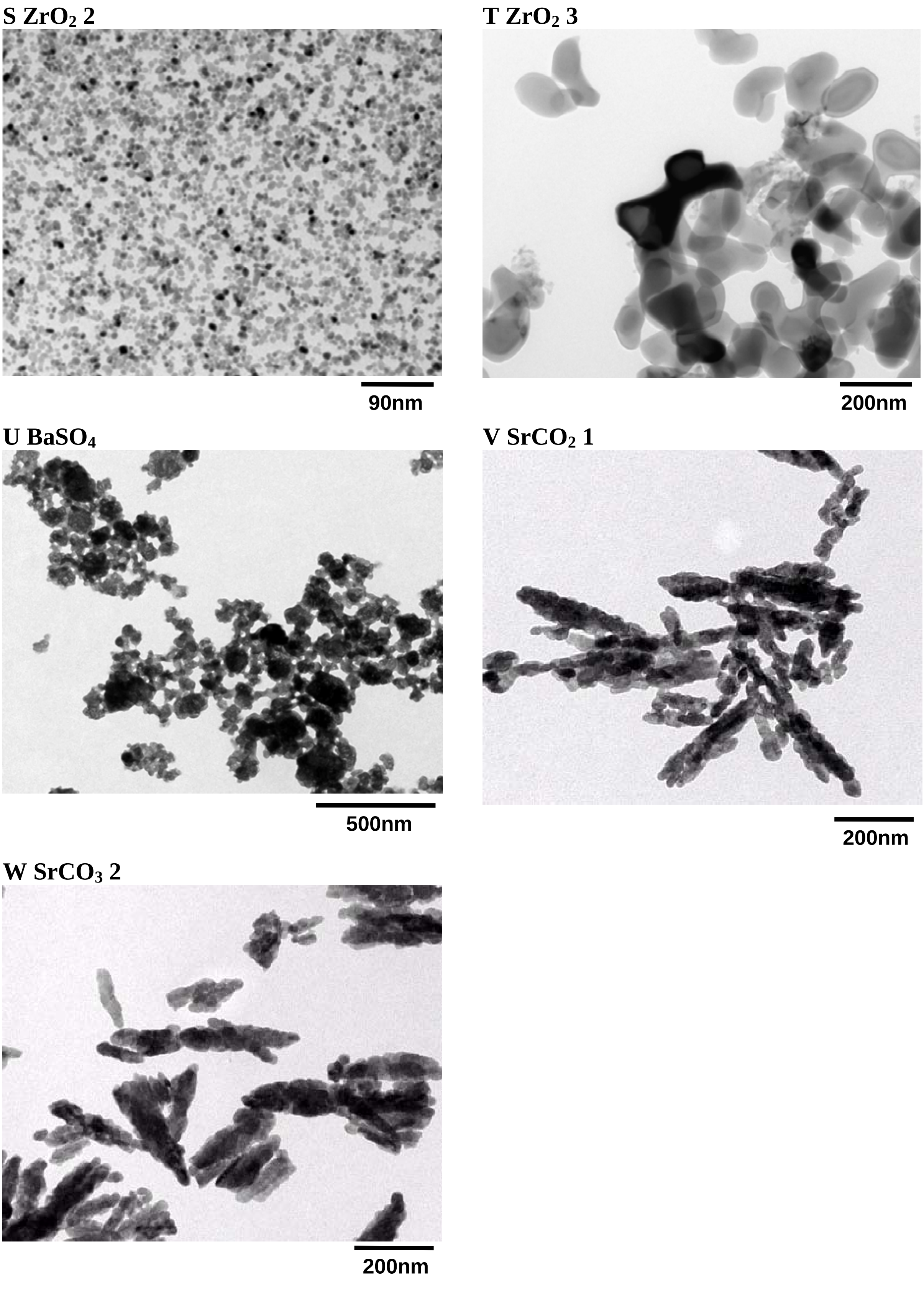

Figure S1. TEM images of the NPs analyzed in this study. A TiO_2 1, B TiO_2 2, C TiO_2 3, D Carbon Black, E CeO, A, F CeO, B, G CeO, C, H CeO, D, I CeO, J AlOOH I, K AlOOH II, L Ti-Zr 1, M Ti-Zr 2, N Ti-Zr 3, O Al-Ti-Zr 1, P Al-Ti-Zr 2, Q Al-Ti-Zr 3, R ZrO_2 1, S ZrO_2 2, T ZrO_2 3, U $BaSO_4$, V $SrCO_3$ 1, W $SrCO_3$. 2 Samples were wetted in ethanol, then gently spread on a sample holder and transferred into vacuum for TEM imaging.

Test procedure standardization

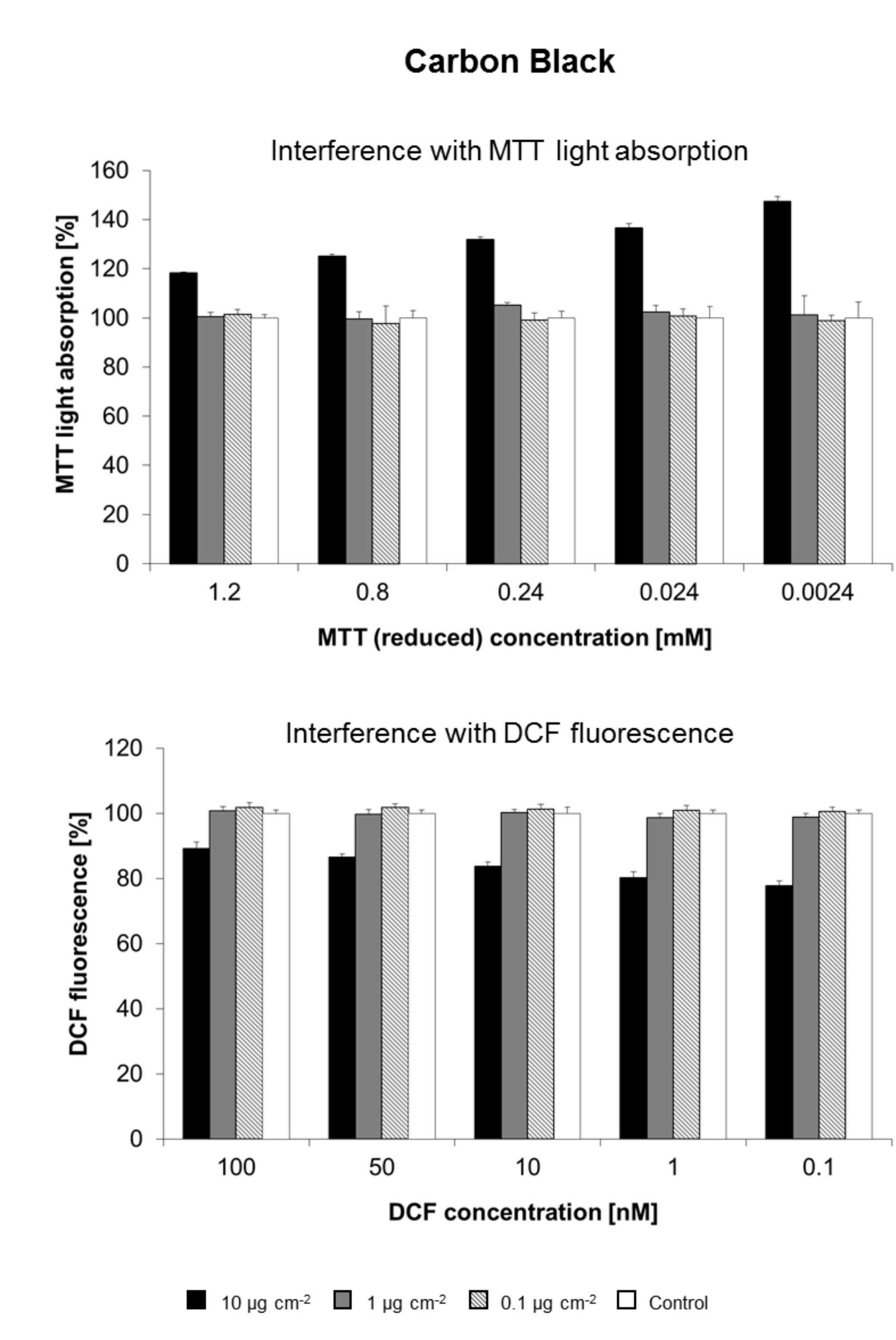

Figure S2. Interference with DCF fluorescence and MTT light absorption by Carbon Black. A549 cells were incubated with different concentrations of Carbon Black in DMEM / 10 % FBS or pure DMEM / 10 % FBS (Control) for 1h (DCF) or 24h (MTT), washed and covered with solutions of oxidized DCF or reduced MTT prior to fluorescence or light absorption measurements (arbitrary units [AU] mean values (n=21, 3x7), with standard deviations).

Table S1. Nanoparticle interference with *in vitro* toxicity test systems: catalytic activity. – no interference detected; + interference detected; ? catalytic activity could not be analyzed due to interference with the optical detection. Empty wells were incubated with NP dispersions in DMEM / 10% FBS or pure DMEM / 10% FBS for 1h (oxidation of DCF) or 24h (reduction of MTT or INT), washed and incubated with H_2DCF-DA or oxidized MTT or INT for 1h (oxidation of DCF), 3h (reduction of MTT) prior to fluorescence/light absorption measurement, or measured continuously for 1h in the presence of oxidized INT according to the assay procedure. DCF fluorescence measurements were taken directly and 3h after incubation with H_2DCF-DA (depicted as directly/3h, e.g. -/-).

		Parameters and particle condition								
		ROS / Oxidative stress			Metabolic activity			Cell death		
		Oxidation of H_2DCF			Reduction of MTT			Reduction of INT		
	**Particles**	**0.1**	**1**	**10**	**0.1**	**1**	**10**	**0.1**	**1**	**10**
1	**TiO_2 1**	-/-	-/-	-/-	-	-	-	-	-	-
2	**TiO_2 2**	-/-	-/-	-/-	-	-	-	-	-	-
3	**TiO_2 3**	-/-	-/-	-/-	-	-	-	-	-	-
4	**Carbon Black**	-/-	-/+	-/+	?	?	?	-	-	-
5	**CeO_2-A**	-/-	-/-	-/-	-	-	-	-	-	-
6	**CeO_2-B**	-/-	-/-	-/-	-	-	-	-	-	-
7	**CeO_2-C**	-/-	-/-	-/-	-	-	-	-	-	-
8	**CeO_2-D**	-/-	-/-	-/-	-	-	-	-	-	-
9	**CeO_2**	-/-	-/-	-/-	-	-	-	-	-	-
10	**AlOOH I**	-/-	-/-	-/-	-	-	-	-	-	-
11	**AlOOH II**	-/-	-/-	-/-	-	-	-	-	-	-
12	**Ti-Zr 1***	-/-	-/-	-/-	-	-	-	-	-	-
13	**Ti-Zr 2***	-/-	-/-	-/-	-	-	-	-	-	-
14	**Ti-Zr 3***	-/-	-/-	-/-	-	-	-	-	-	-
15	**Al-Ti-Zr 1***	-/-	-/-	-/-	-	-	-	-	-	-
16	**Al-Ti-Zr 2***	-/-	-/-	-/-	-	-	-	-	-	-
17	**Al-Ti-Zr 3***	-/-	-/-	-/-	-	-	-	-	-	-
18	**ZrO_2 1**	-/-	-/-	-/-	-	-	-	-	-	-
19	**ZrO_2 2**	-/-	-/-	-/-	-	-	-	-	-	-
20	**ZrO_2 3**	-/-	-/-	-/-	-	-	-	-	-	-
21	**$BaSO_4$**	-/-	-/-	-/-	-	-	-	-	-	-
22	**$SrCO_3$ 1**	-/-	-/-	-/-	-	-	-	-	-	-
23	**$SrCO_3$ 2**	-/-	-/-	-/-	-	-	-	-	-	-

In vitro **toxicity screenings**

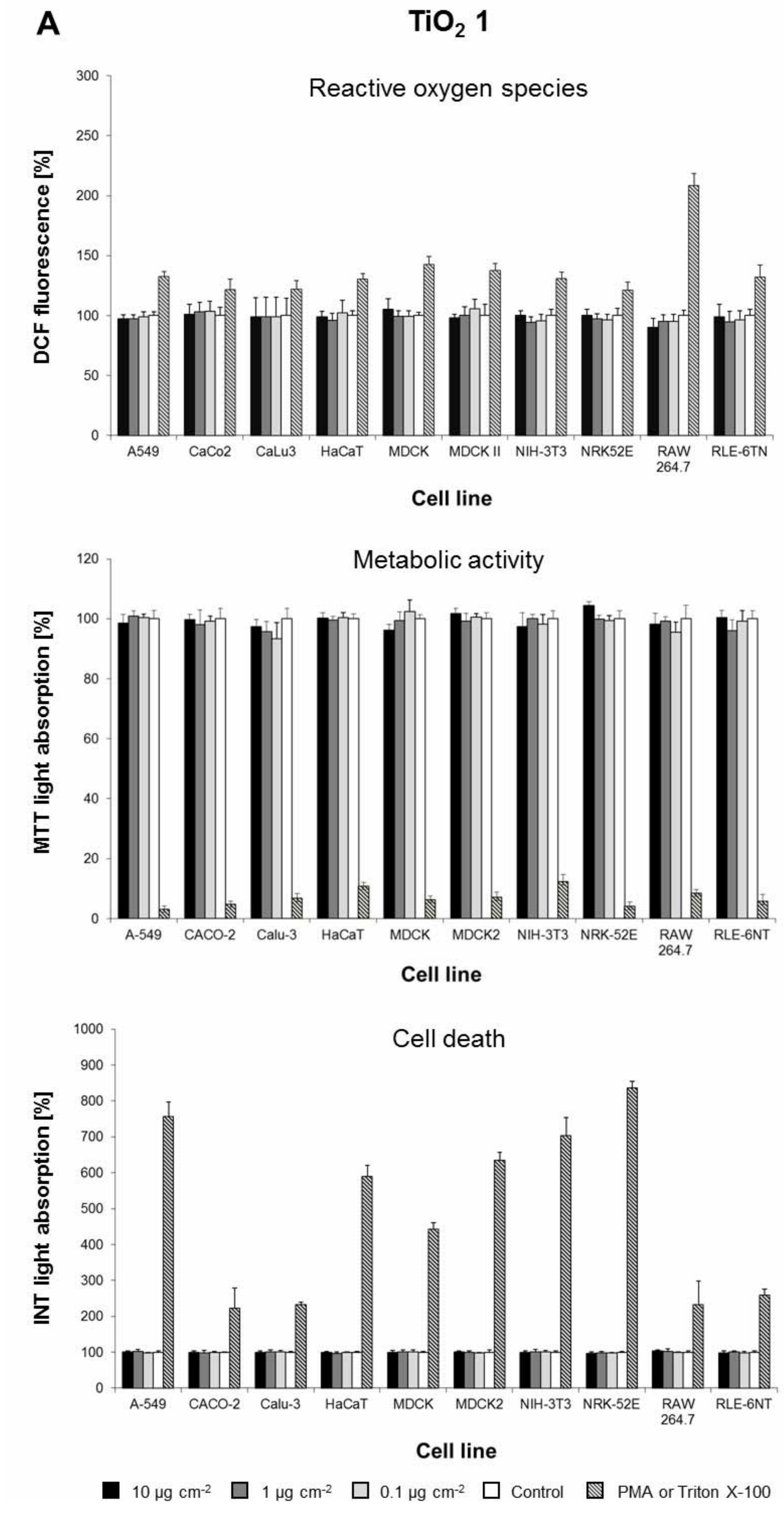

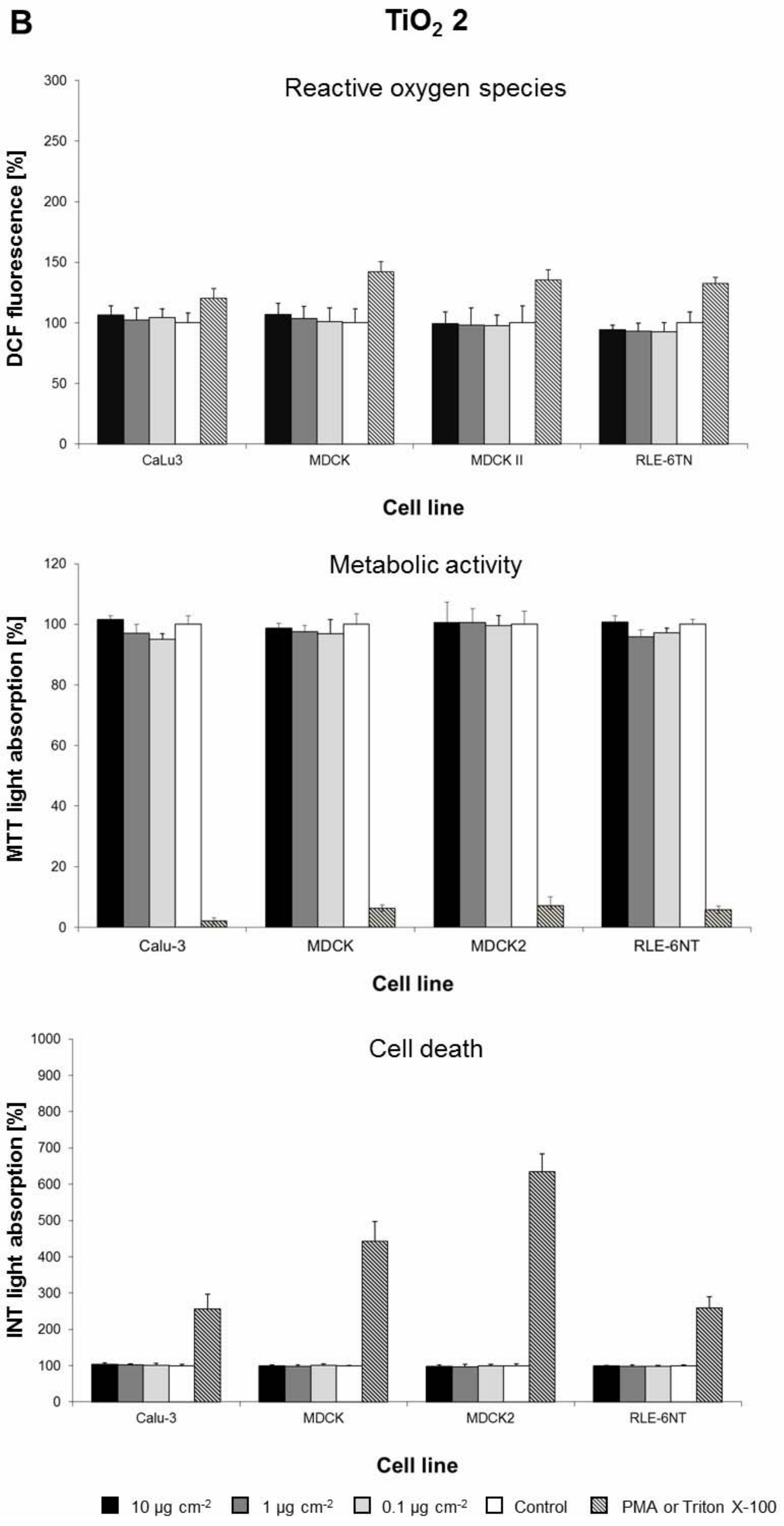
B
TiO2 2
Reactive oxygen species
DCF fluorescence [%]
300
250
200
150
100
50
0
CaLu3
MDCK
MDCK II
RLE-6TN
Cell line
Metabolic activity
MTT light absorption [%]
120
100
80
60
40
20
0
Calu-3
MDCK
MDCK2
RLE-6NT
Cell line
Cell death
INT light absorption [%]
1000
900
800
700
600
500
400
300
200
100
0
Calu-3
MDCK
MDCK2
RLE-6NT
Cell line
10 µg cm-2
1 µg cm-2
0.1 µg cm-2
Control
PMA or Triton X-100

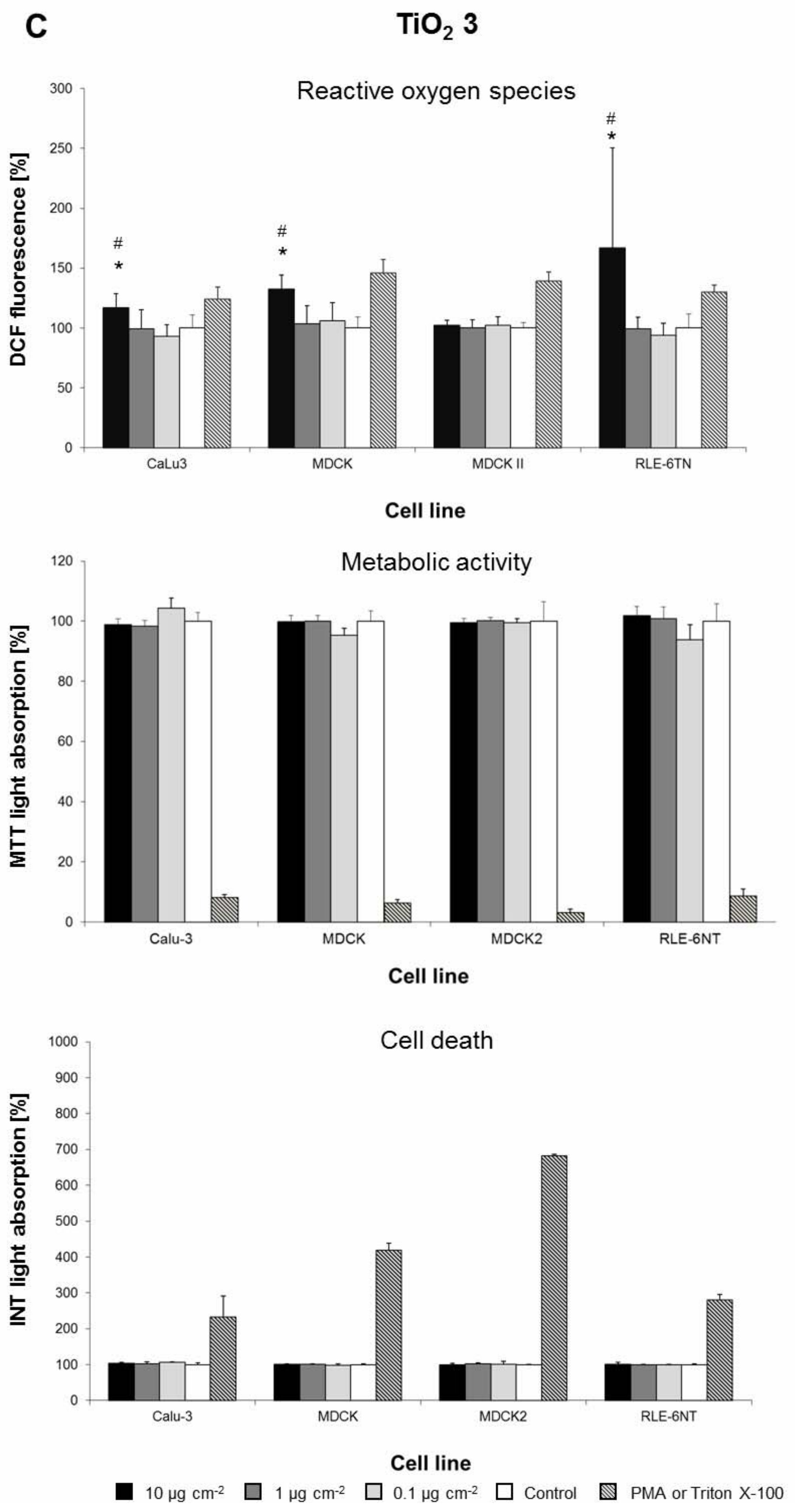
C
TiO2 3
Reactive oxygen species
DCF fluorescence [%]
300
250
200
150
100
50
0
#
*
CaLu3
MDCK
MDCK II
RLE-6TN
Cell line
Metabolic activity
MTT light absorption [%]
120
100
80
60
40
20
0
Calu-3
MDCK
MDCK2
RLE-6NT
Cell line
Cell death
INT light absorption [%]
1000
900
800
700
600
500
400
300
200
100
0
Calu-3
MDCK
MDCK2
RLE-6NT
Cell line
10 µg cm-2
1 µg cm-2
0.1 µg cm-2
Control
PMA or Triton X-100

D **Carbon Black**

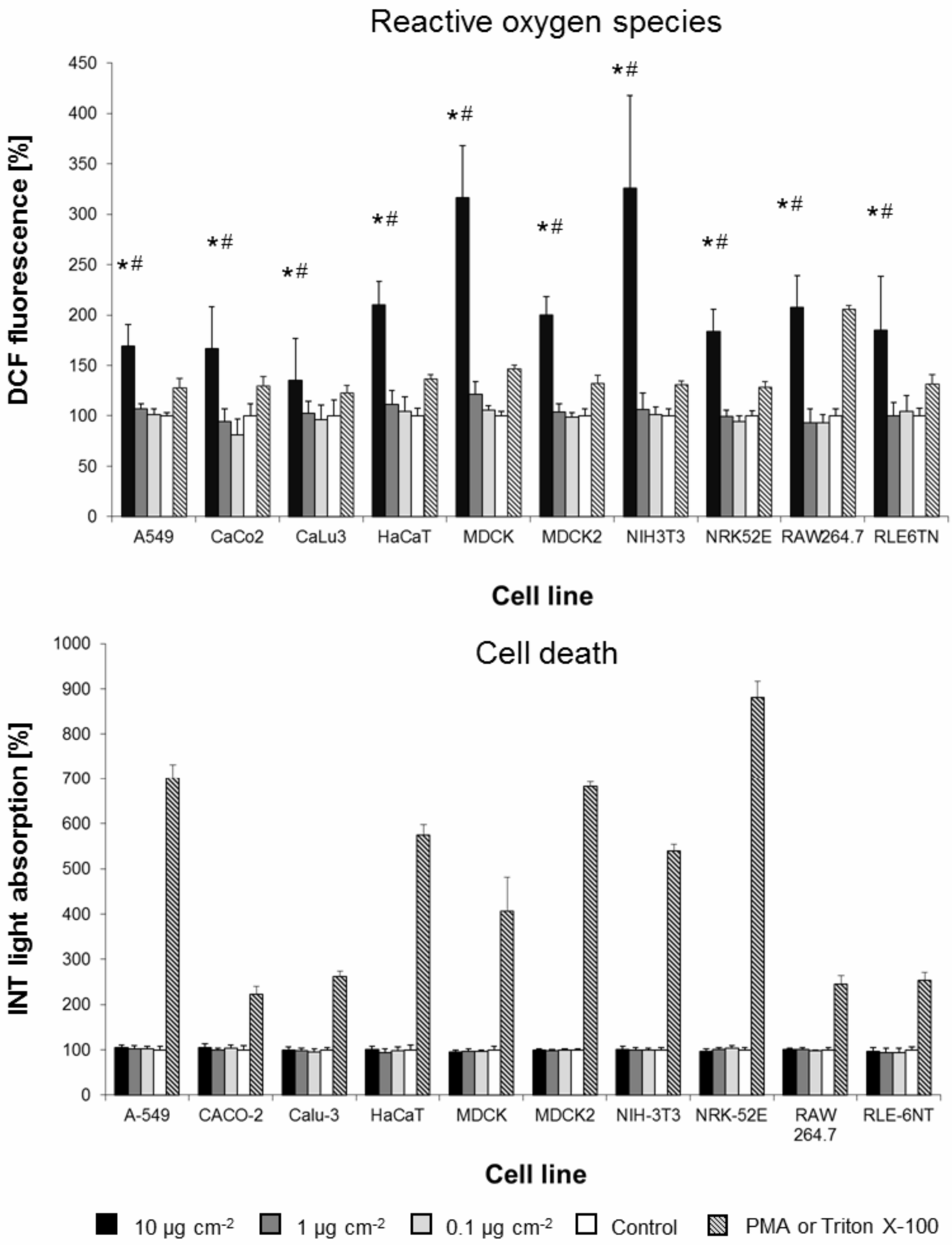

E

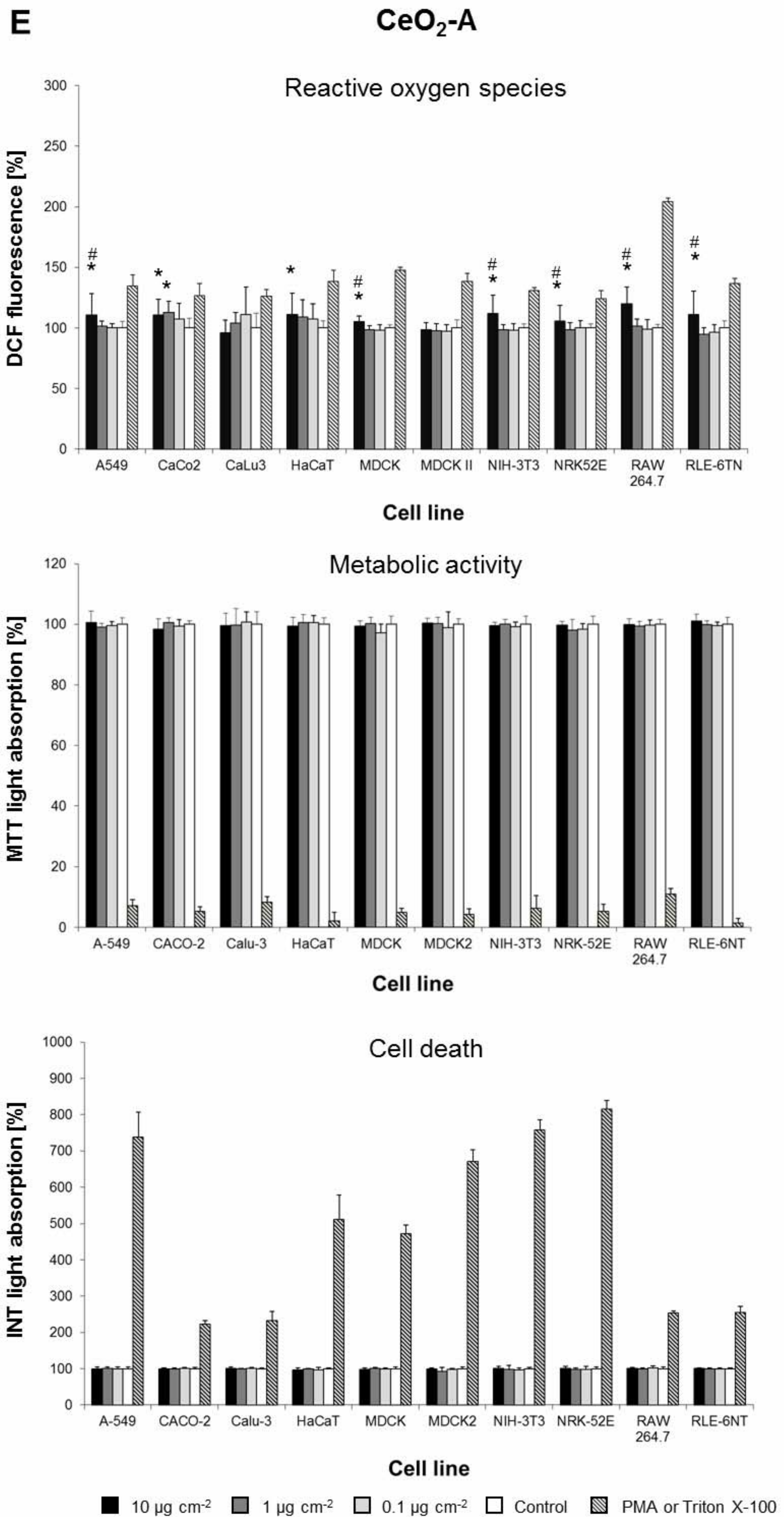

CeO2-A
Reactive oxygen species
DCF fluorescence [%]
300
250
200
150
100
50
0
A549
CaCo2
CaLu3
HaCaT
MDCK
MDCK II
NIH-3T3
NRK52E
RAW 264.7
RLE-6TN
Cell line
Metabolic activity
MTT light absorption [%]
120
100
80
60
40
20
0
A-549
CACO-2
Calu-3
HaCaT
MDCK
MDCK2
NIH-3T3
NRK-52E
RAW 264.7
RLE-6NT
Cell line
Cell death
INT light absorption [%]
1000
900
800
700
600
500
400
300
200
100
0
A-549
CACO-2
Calu-3
HaCaT
MDCK
MDCK2
NIH-3T3
NRK-52E
RAW 264.7
RLE-6NT
Cell line
10 µg cm-2
1 µg cm-2
0.1 µg cm-2
Control
PMA or Triton X-100

F

CeO_2-B

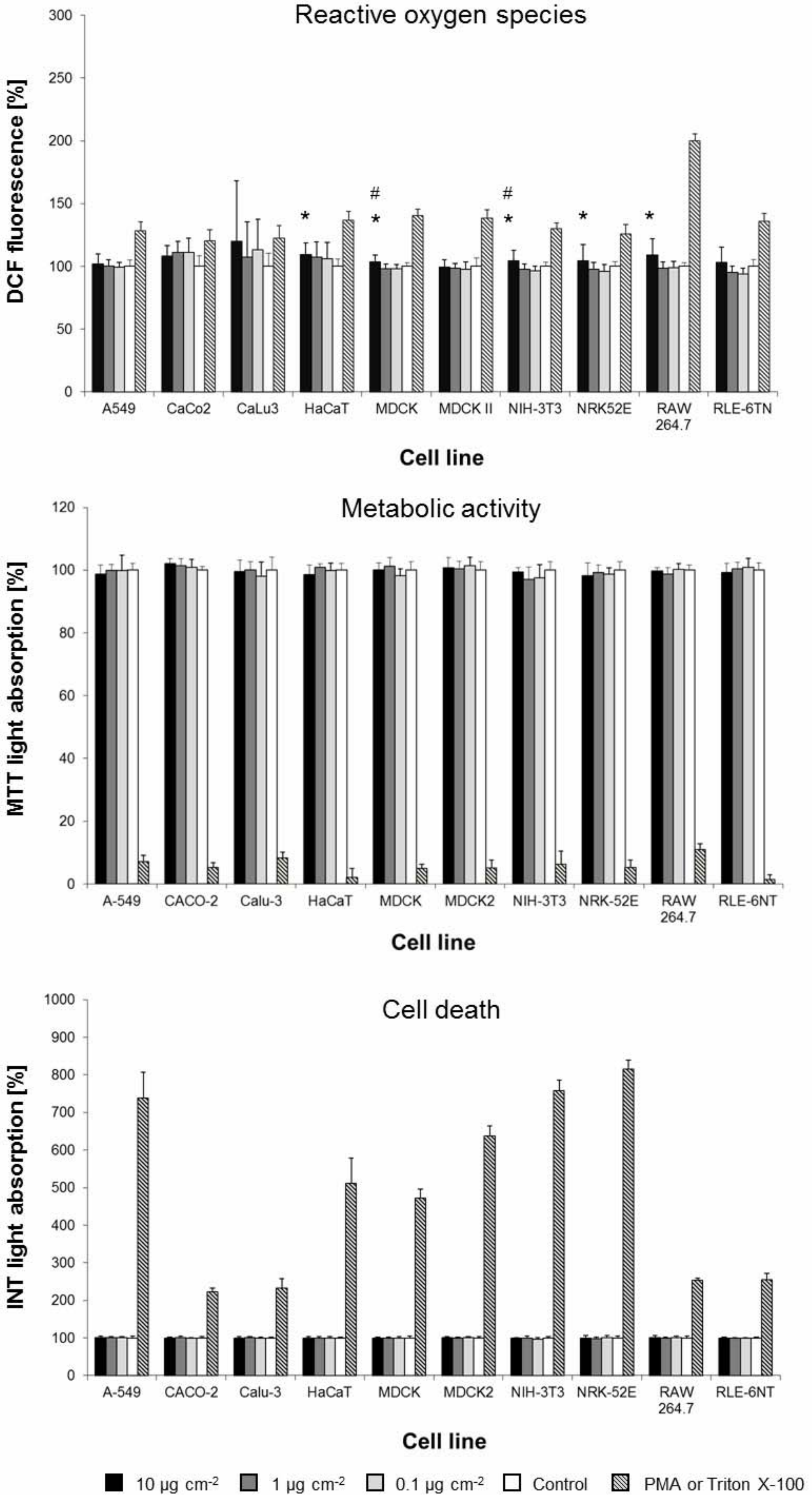
Reactive oxygen species
DCF fluorescence [%]
A549
CaCo2
CaLu3
HaCaT
MDCK
MDCK II
NIH-3T3
NRK52E
RAW 264.7
RLE-6TN
Cell line
Metabolic activity
MTT light absorption [%]
A-549
CACO-2
Calu-3
HaCaT
MDCK
MDCK2
NIH-3T3
NRK-52E
RAW 264.7
RLE-6NT
Cell line
Cell death
INT light absorption [%]
A-549
CACO-2
Calu-3
HaCaT
MDCK
MDCK2
NIH-3T3
NRK-52E
RAW 264.7
RLE-6NT
Cell line
10 µg cm-2
1 µg cm-2
0.1 µg cm-2
Control
PMA or Triton X-100

G

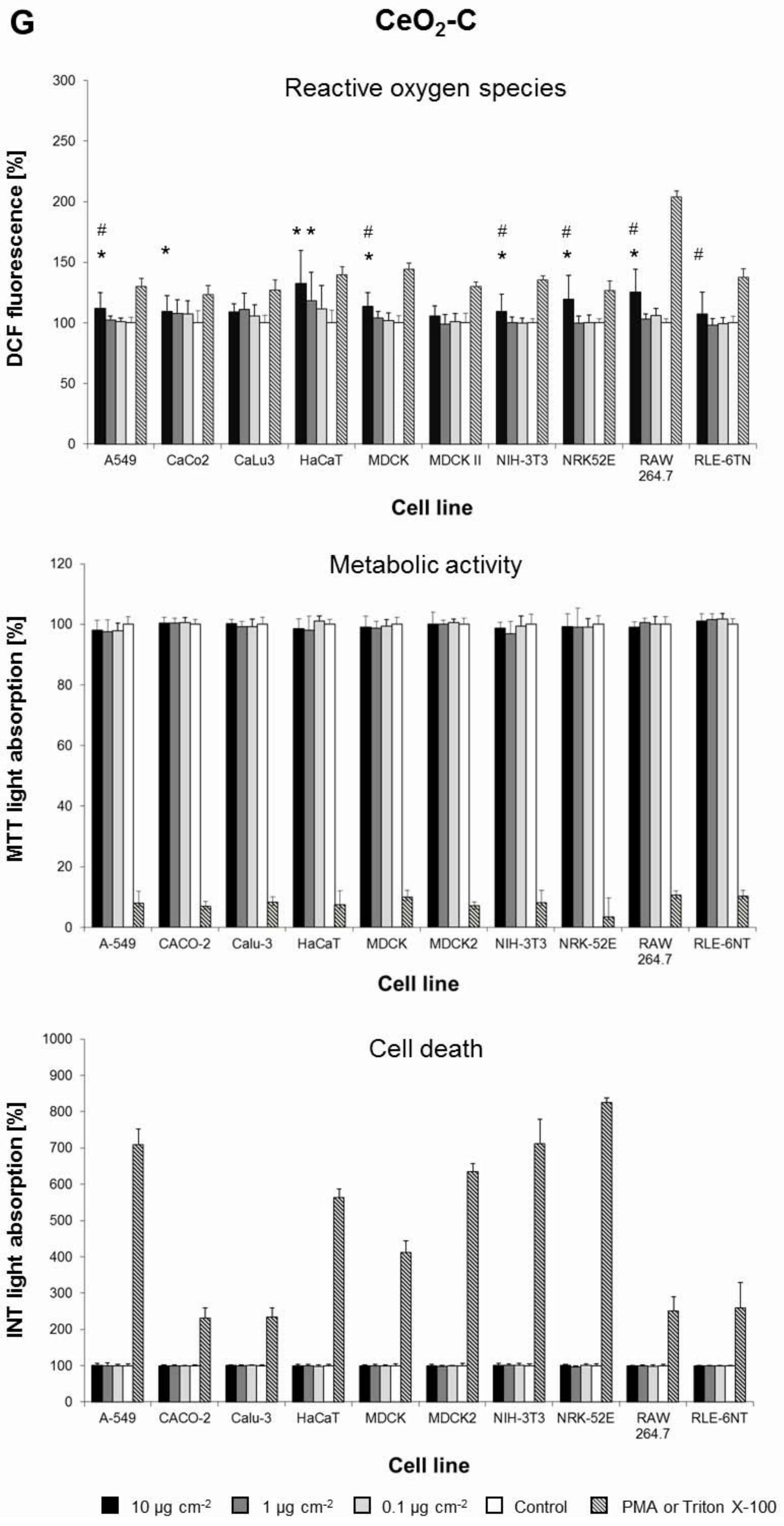

CeO2-C
Reactive oxygen species
DCF fluorescence [%]
300
250
200
150
100
50
0
A549
CaCo2
CaLu3
HaCaT
MDCK
MDCK II
NIH-3T3
NRK52E
RAW 264.7
RLE-6TN
Cell line
Metabolic activity
MTT light absorption [%]
120
100
80
60
40
20
0
A-549
CACO-2
Calu-3
HaCaT
MDCK
MDCK2
NIH-3T3
NRK-52E
RAW 264.7
RLE-6NT
Cell line
Cell death
INT light absorption [%]
1000
900
800
700
600
500
400
300
200
100
0
A-549
CACO-2
Calu-3
HaCaT
MDCK
MDCK2
NIH-3T3
NRK-52E
RAW 264.7
RLE-6NT
Cell line
10 µg cm-2
1 µg cm-2
0.1 µg cm-2
Control
PMA or Triton X-100

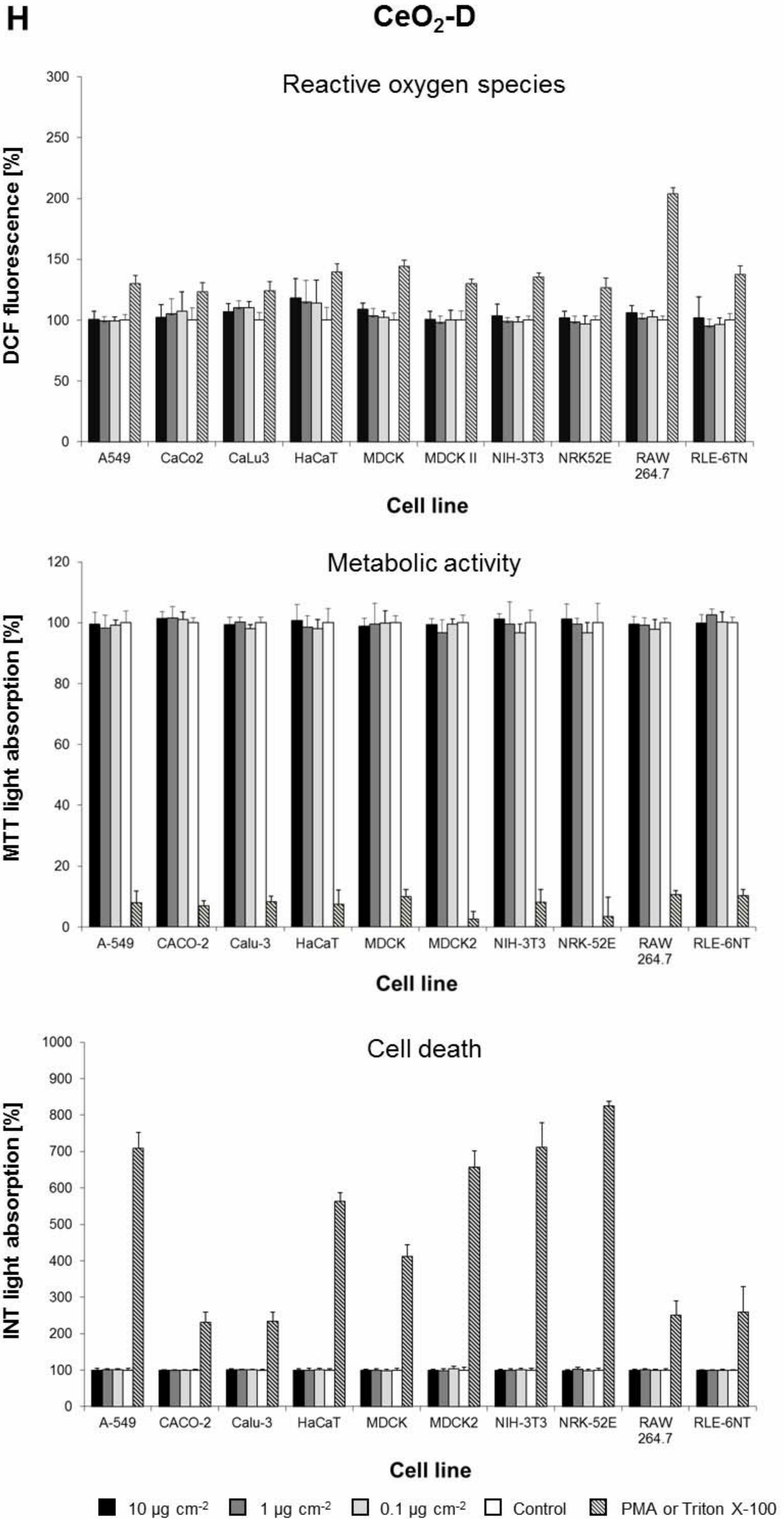
H
CeO2-D
Reactive oxygen species
DCF fluorescence [%]
0
50
100
150
200
250
300
A549
CaCo2
CaLu3
HaCaT
MDCK
MDCK II
NIH-3T3
NRK52E
RAW 264.7
RLE-6TN
Cell line
Metabolic activity
MTT light absorption [%]
0
20
40
60
80
100
120
A-549
CACO-2
Calu-3
HaCaT
MDCK
MDCK2
NIH-3T3
NRK-52E
RAW 264.7
RLE-6NT
Cell line
Cell death
INT light absorption [%]
0
100
200
300
400
500
600
700
800
900
1000
A-549
CACO-2
Calu-3
HaCaT
MDCK
MDCK2
NIH-3T3
NRK-52E
RAW 264.7
RLE-6NT
Cell line
10 µg cm-2
1 µg cm-2
0.1 µg cm-2
Control
PMA or Triton X-100

I

CeO_2

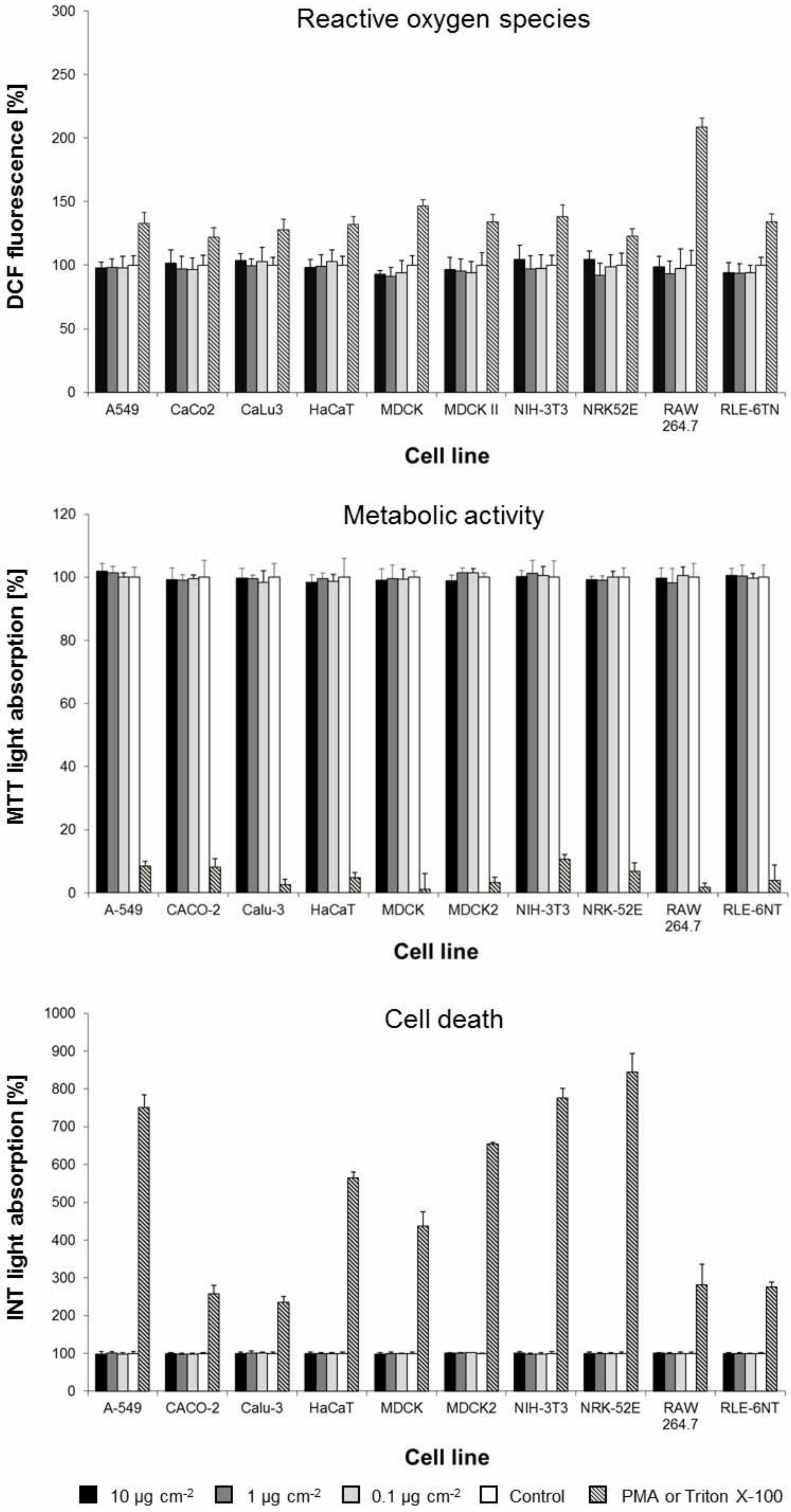
Reactive oxygen species
DCF fluorescence [%]
300
250
200
150
100
50
0
A549
CaCo2
CaLu3
HaCaT
MDCK
MDCK II
NIH-3T3
NRK52E
RAW 264.7
RLE-6TN
Cell line
Metabolic activity
MTT light absorption [%]
120
100
80
60
40
20
0
A-549
CACO-2
Calu-3
HaCaT
MDCK
MDCK2
NIH-3T3
NRK-52E
RAW 264.7
RLE-6NT
Cell line
Cell death
INT light absorption [%]
1000
900
800
700
600
500
400
300
200
100
0
A-549
CACO-2
Calu-3
HaCaT
MDCK
MDCK2
NIH-3T3
NRK-52E
RAW 264.7
RLE-6NT
Cell line
10 µg cm-2
1 µg cm-2
0.1 µg cm-2
Control
PMA or Triton X-100

J

AlOOH I

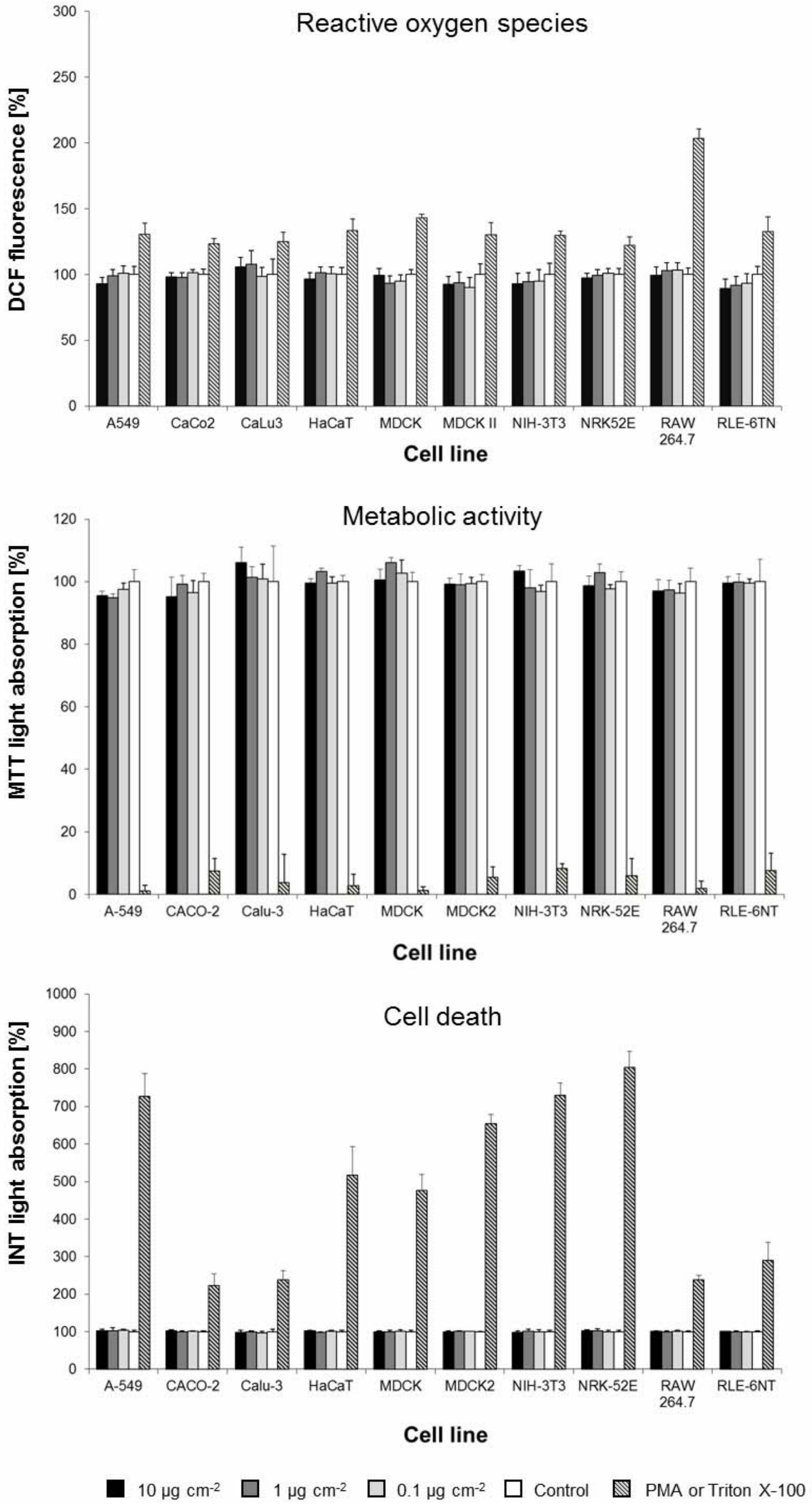
Reactive oxygen species
DCF fluorescence [%]
0
50
100
150
200
250
300
A549
CaCo2
CaLu3
HaCaT
MDCK
MDCK II
NIH-3T3
NRK52E
RAW 264.7
RLE-6TN
Cell line
Metabolic activity
MTT light absorption [%]
0
20
40
60
80
100
120
A-549
CACO-2
Calu-3
HaCaT
MDCK
MDCK2
NIH-3T3
NRK-52E
RAW 264.7
RLE-6NT
Cell line
Cell death
INT light absorption [%]
0
100
200
300
400
500
600
700
800
900
1000
A-549
CACO-2
Calu-3
HaCaT
MDCK
MDCK2
NIH-3T3
NRK-52E
RAW 264.7
RLE-6NT
Cell line
10 µg cm-2
1 µg cm-2
0.1 µg cm-2
Control
PMA or Triton X-100

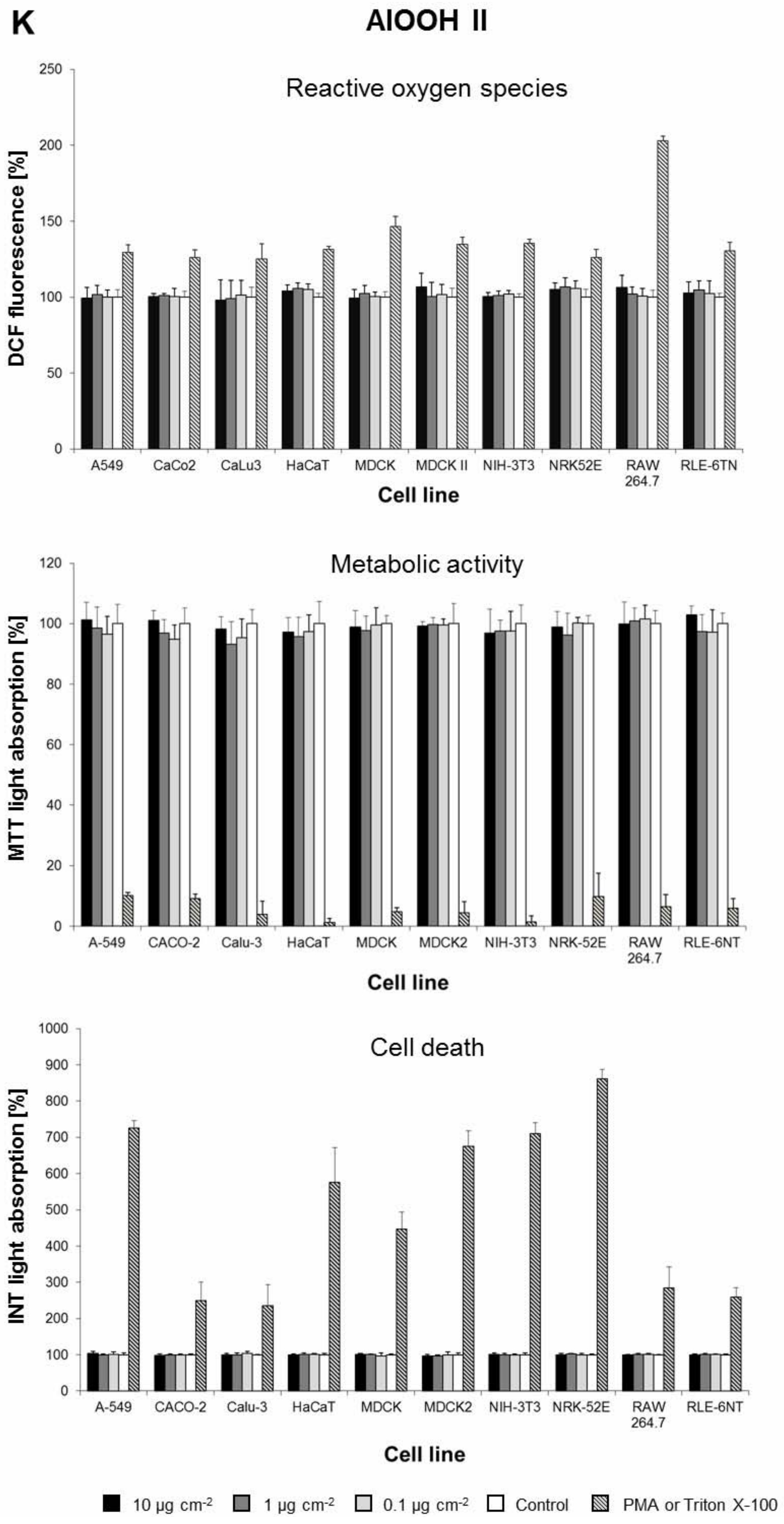
K
AlOOH II
Reactive oxygen species
DCF fluorescence [%]
0
50
100
150
200
250
A549
CaCo2
CaLu3
HaCaT
MDCK
MDCK II
NIH-3T3
NRK52E
RAW 264.7
RLE-6TN
Cell line
Metabolic activity
MTT light absorption [%]
0
20
40
60
80
100
120
A-549
CACO-2
Calu-3
HaCaT
MDCK
MDCK2
NIH-3T3
NRK-52E
RAW 264.7
RLE-6NT
Cell line
Cell death
INT light absorption [%]
0
100
200
300
400
500
600
700
800
900
1000
A-549
CACO-2
Calu-3
HaCaT
MDCK
MDCK2
NIH-3T3
NRK-52E
RAW 264.7
RLE-6NT
Cell line
10 µg cm-2
1 µg cm-2
0.1 µg cm-2
Control
PMA or Triton X-100

L

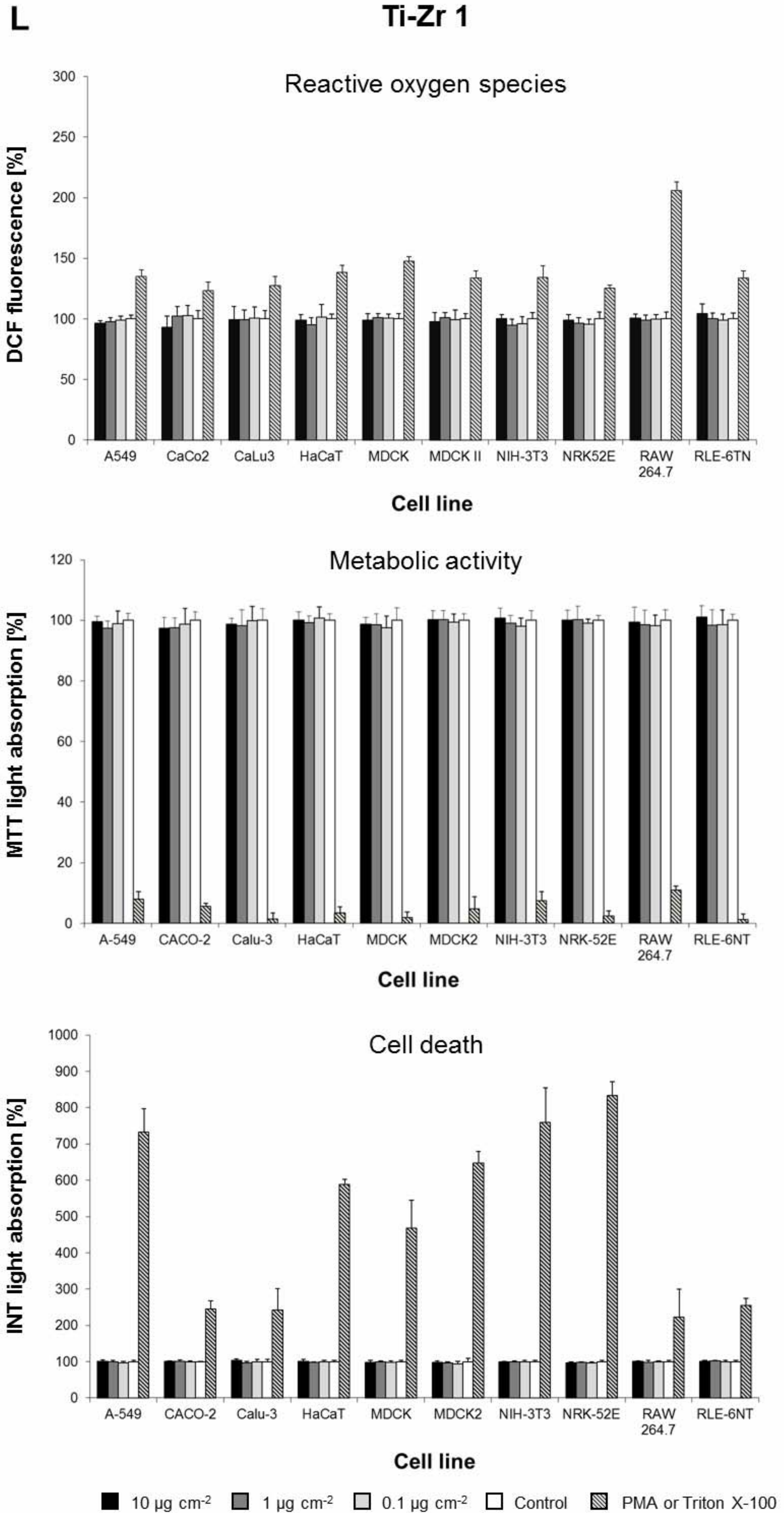
Ti-Zr 1
Reactive oxygen species
DCF fluorescence [%]
300
250
200
150
100
50
0
A549
CaCo2
CaLu3
HaCaT
MDCK
MDCK II
NIH-3T3
NRK52E
RAW 264.7
RLE-6TN
Cell line
Metabolic activity
MTT light absorption [%]
120
100
80
60
40
20
0
A-549
CACO-2
Calu-3
HaCaT
MDCK
MDCK2
NIH-3T3
NRK-52E
RAW 264.7
RLE-6NT
Cell line
Cell death
INT light absorption [%]
1000
900
800
700
600
500
400
300
200
100
0
A-549
CACO-2
Calu-3
HaCaT
MDCK
MDCK2
NIH-3T3
NRK-52E
RAW 264.7
RLE-6NT
Cell line
10 µg cm-2
1 µg cm-2
0.1 µg cm-2
Control
PMA or Triton X-100

M

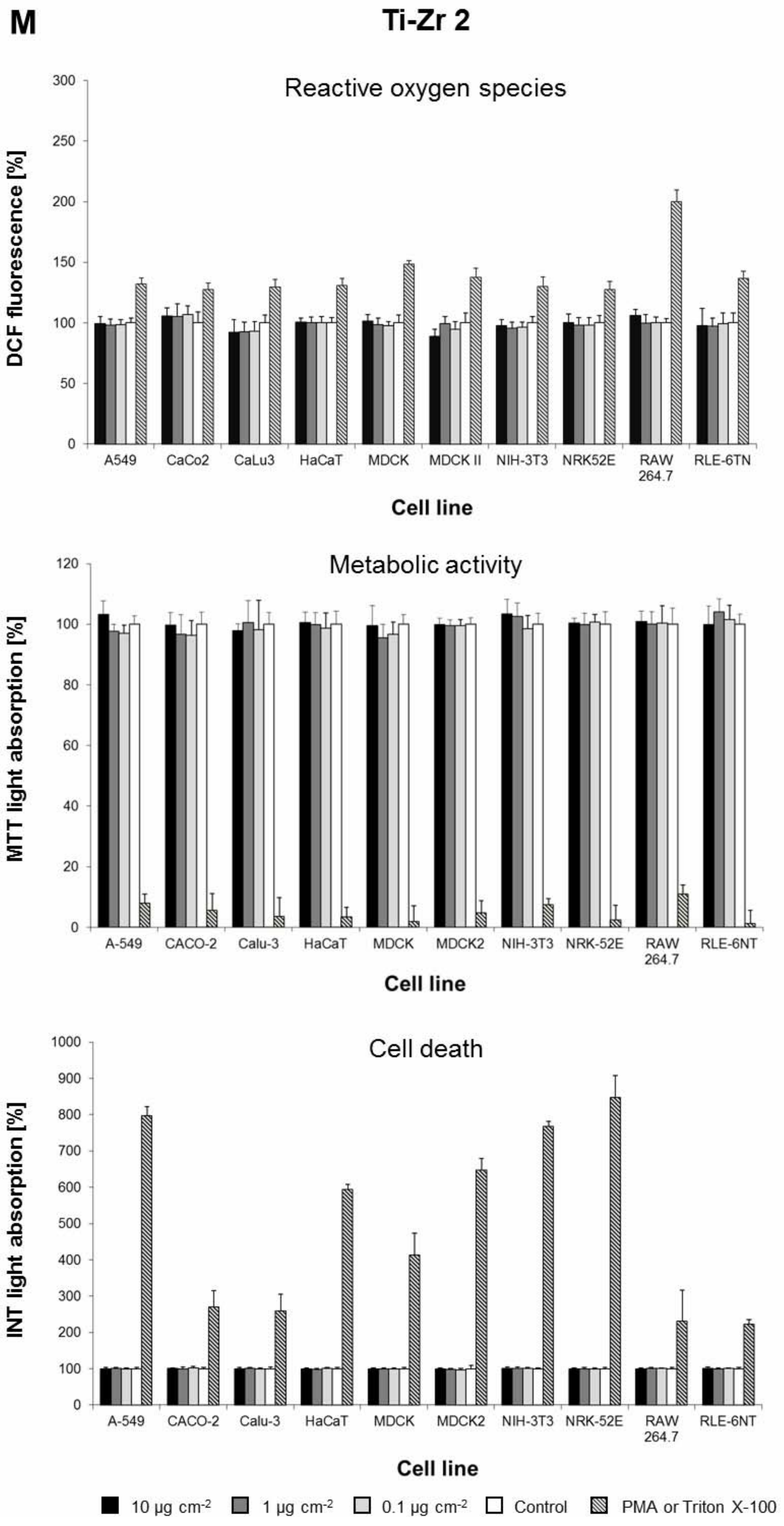

Ti-Zr 2
Reactive oxygen species
DCF fluorescence [%]
300
250
200
150
100
50
0
A549
CaCo2
CaLu3
HaCaT
MDCK
MDCK II
NIH-3T3
NRK52E
RAW 264.7
RLE-6TN
Cell line
Metabolic activity
MTT light absorption [%]
120
100
80
60
40
20
0
A-549
CACO-2
Calu-3
HaCaT
MDCK
MDCK2
NIH-3T3
NRK-52E
RAW 264.7
RLE-6NT
Cell line
Cell death
INT light absorption [%]
1000
900
800
700
600
500
400
300
200
100
0
A-549
CACO-2
Calu-3
HaCaT
MDCK
MDCK2
NIH-3T3
NRK-52E
RAW 264.7
RLE-6NT
Cell line
10 µg cm-2
1 µg cm-2
0.1 µg cm-2
Control
PMA or Triton X-100

N **Ti-Zr 3**

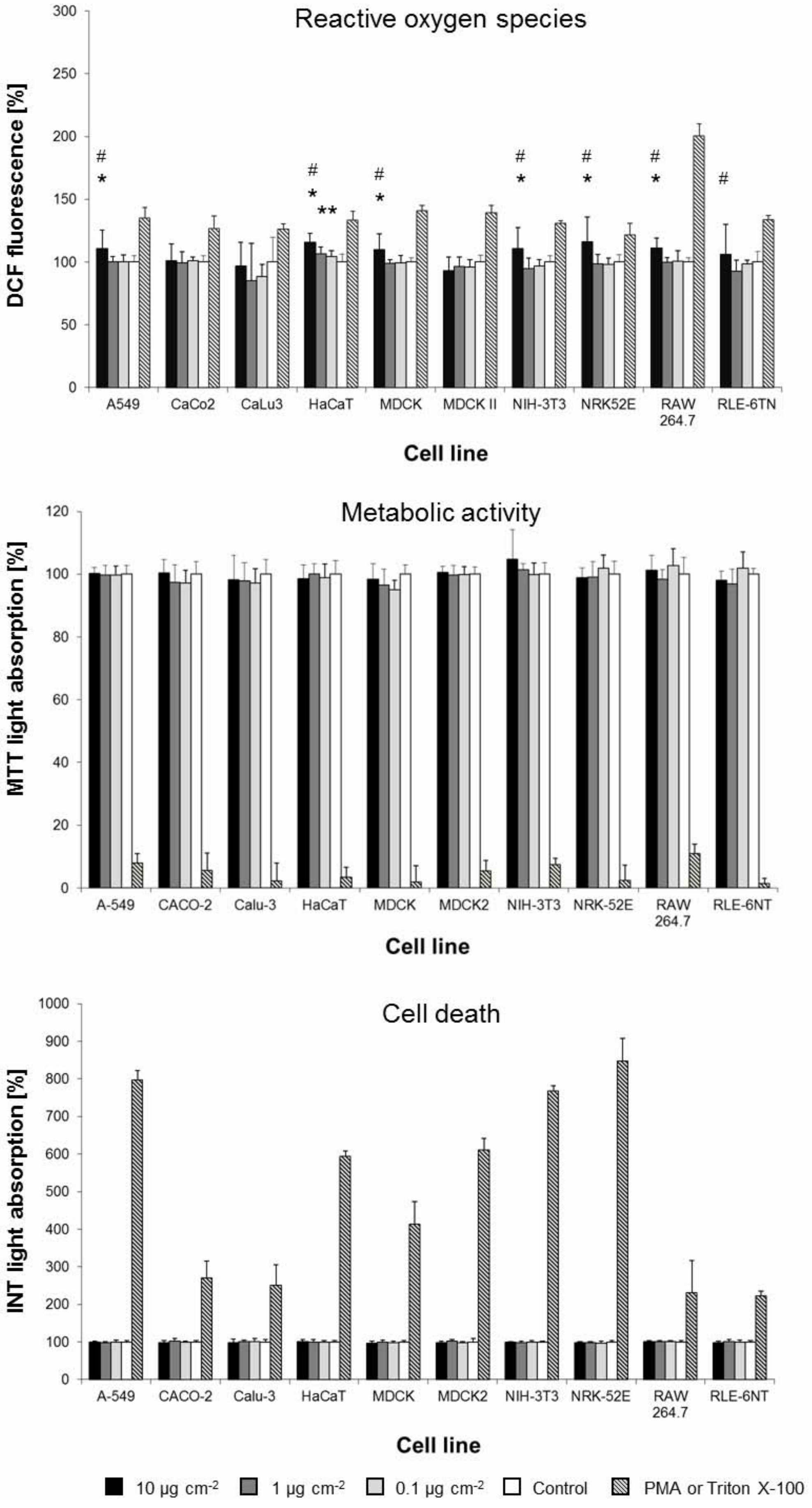
Reactive oxygen species
DCF fluorescence [%]
0
50
100
150
200
250
300
#
*
**
A549
CaCo2
CaLu3
HaCaT
MDCK
MDCK II
NIH-3T3
NRK52E
RAW 264.7
RLE-6TN
Cell line
Metabolic activity
MTT light absorption [%]
0
20
40
60
80
100
120
A-549
CACO-2
Calu-3
HaCaT
MDCK
MDCK2
NIH-3T3
NRK-52E
RAW 264.7
RLE-6NT
Cell line
Cell death
INT light absorption [%]
0
100
200
300
400
500
600
700
800
900
1000
A-549
CACO-2
Calu-3
HaCaT
MDCK
MDCK2
NIH-3T3
NRK-52E
RAW 264.7
RLE-6NT
Cell line
10 µg cm-2
1 µg cm-2
0.1 µg cm-2
Control
PMA or Triton X-100

O

Al-Ti-Zr 1

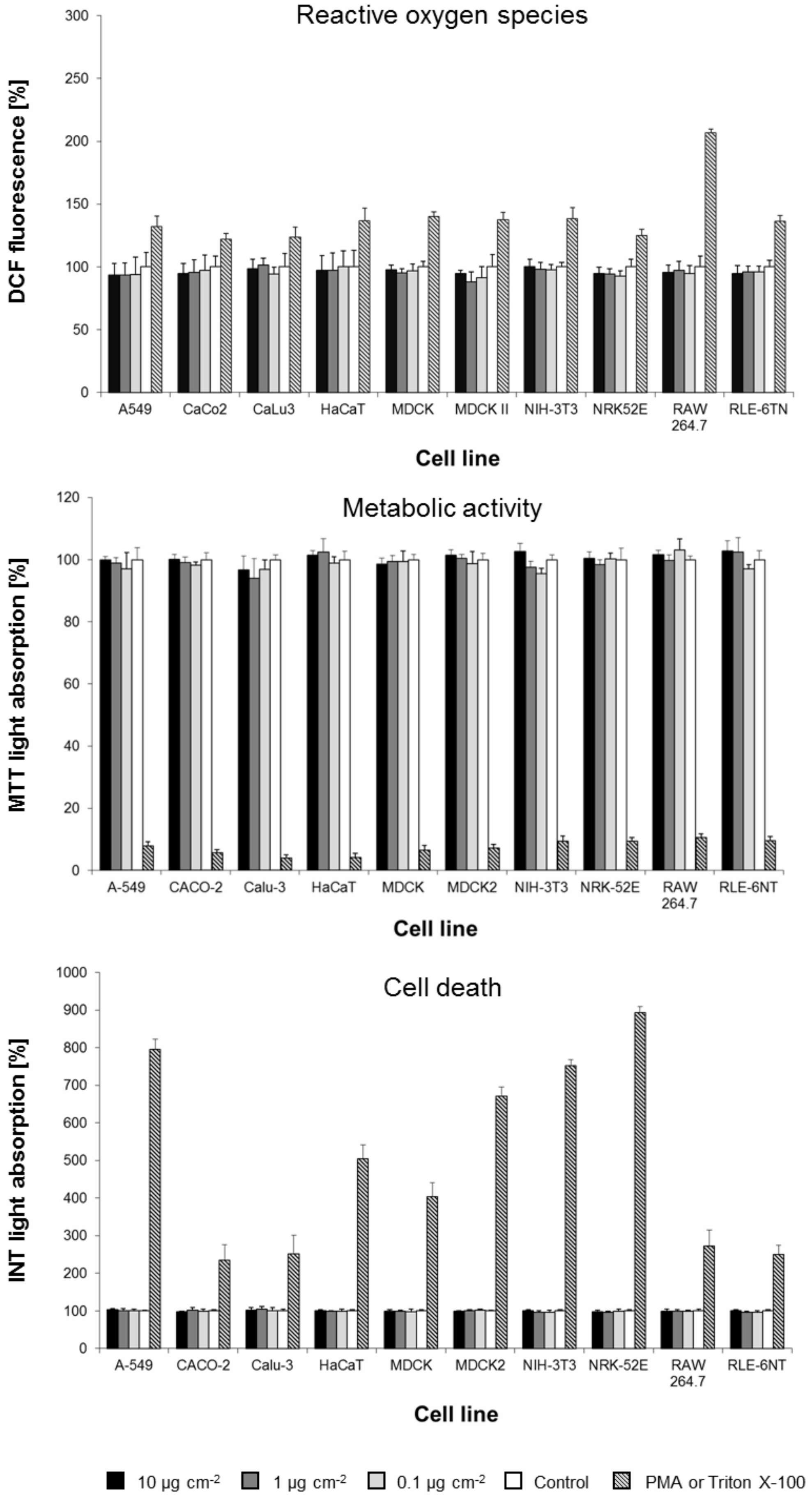

Reactive oxygen species
DCF fluorescence [%]
300
250
200
150
100
50
0
A549
CaCo2
CaLu3
HaCaT
MDCK
MDCK II
NIH-3T3
NRK52E
RAW 264.7
RLE-6TN
Cell line
Metabolic activity
MTT light absorption [%]
120
100
80
60
40
20
0
A-549
CACO-2
Calu-3
HaCaT
MDCK
MDCK2
NIH-3T3
NRK-52E
RAW 264.7
RLE-6NT
Cell line
Cell death
INT light absorption [%]
1000
900
800
700
600
500
400
300
200
100
0
A-549
CACO-2
Calu-3
HaCaT
MDCK
MDCK2
NIH-3T3
NRK-52E
RAW 264.7
RLE-6NT
Cell line
10 µg cm-2
1 µg cm-2
0.1 µg cm-2
Control
PMA or Triton X-100

P

Al-Ti-Zr 2

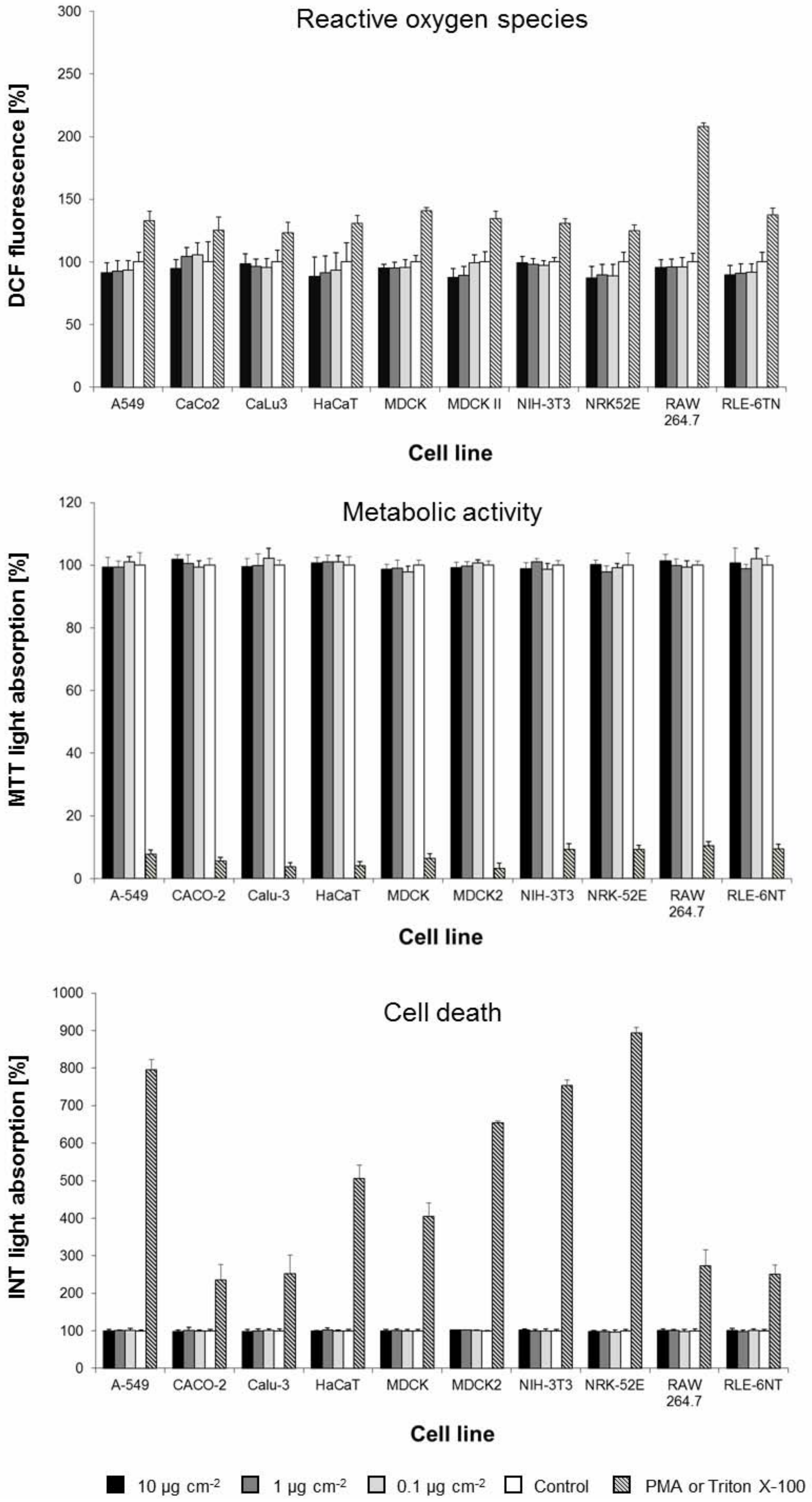

Reactive oxygen species
DCF fluorescence [%]
300
250
200
150
100
50
0
A549
CaCo2
CaLu3
HaCaT
MDCK
MDCK II
NIH-3T3
NRK52E
RAW 264.7
RLE-6TN
Cell line
Metabolic activity
MTT light absorption [%]
120
100
80
60
40
20
0
A-549
CACO-2
Calu-3
HaCaT
MDCK
MDCK2
NIH-3T3
NRK-52E
RAW 264.7
RLE-6NT
Cell line
Cell death
INT light absorption [%]
1000
900
800
700
600
500
400
300
200
100
0
A-549
CACO-2
Calu-3
HaCaT
MDCK
MDCK2
NIH-3T3
NRK-52E
RAW 264.7
RLE-6NT
Cell line
10 µg cm-2
1 µg cm-2
0.1 µg cm-2
Control
PMA or Triton X-100

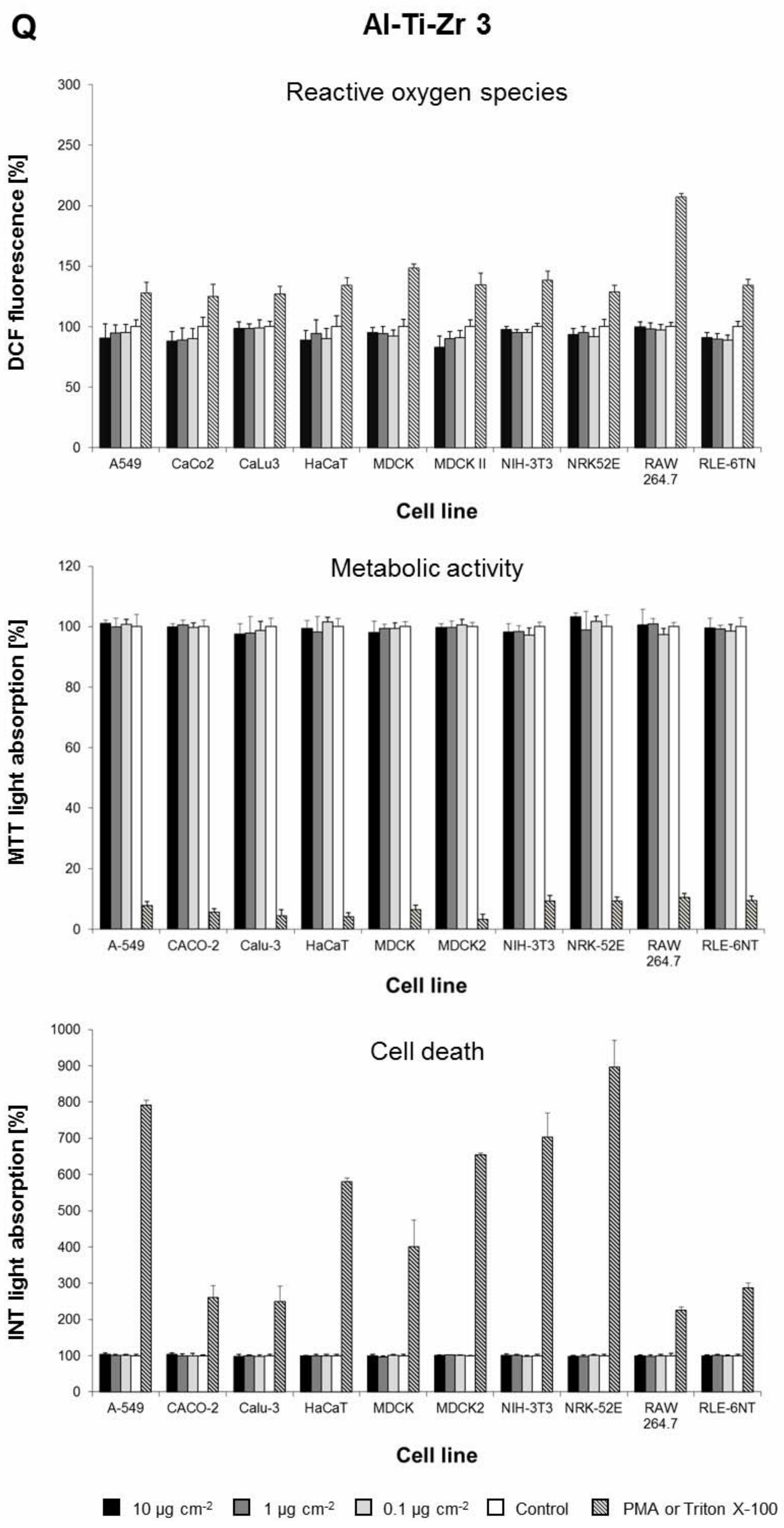

Q
Al-Ti-Zr 3
Reactive oxygen species
DCF fluorescence [%]
300
250
200
150
100
50
0
A549
CaCo2
CaLu3
HaCaT
MDCK
MDCK II
NIH-3T3
NRK52E
RAW 264.7
RLE-6TN
Cell line
Metabolic activity
MTT light absorption [%]
120
100
80
60
40
20
0
A-549
CACO-2
Calu-3
HaCaT
MDCK
MDCK2
NIH-3T3
NRK-52E
RAW 264.7
RLE-6NT
Cell line
Cell death
INT light absorption [%]
1000
900
800
700
600
500
400
300
200
100
0
A-549
CACO-2
Calu-3
HaCaT
MDCK
MDCK2
NIH-3T3
NRK-52E
RAW 264.7
RLE-6NT
Cell line
10 µg cm-2
1 µg cm-2
0.1 µg cm-2
Control
PMA or Triton X-100

R **ZrO_2 1**

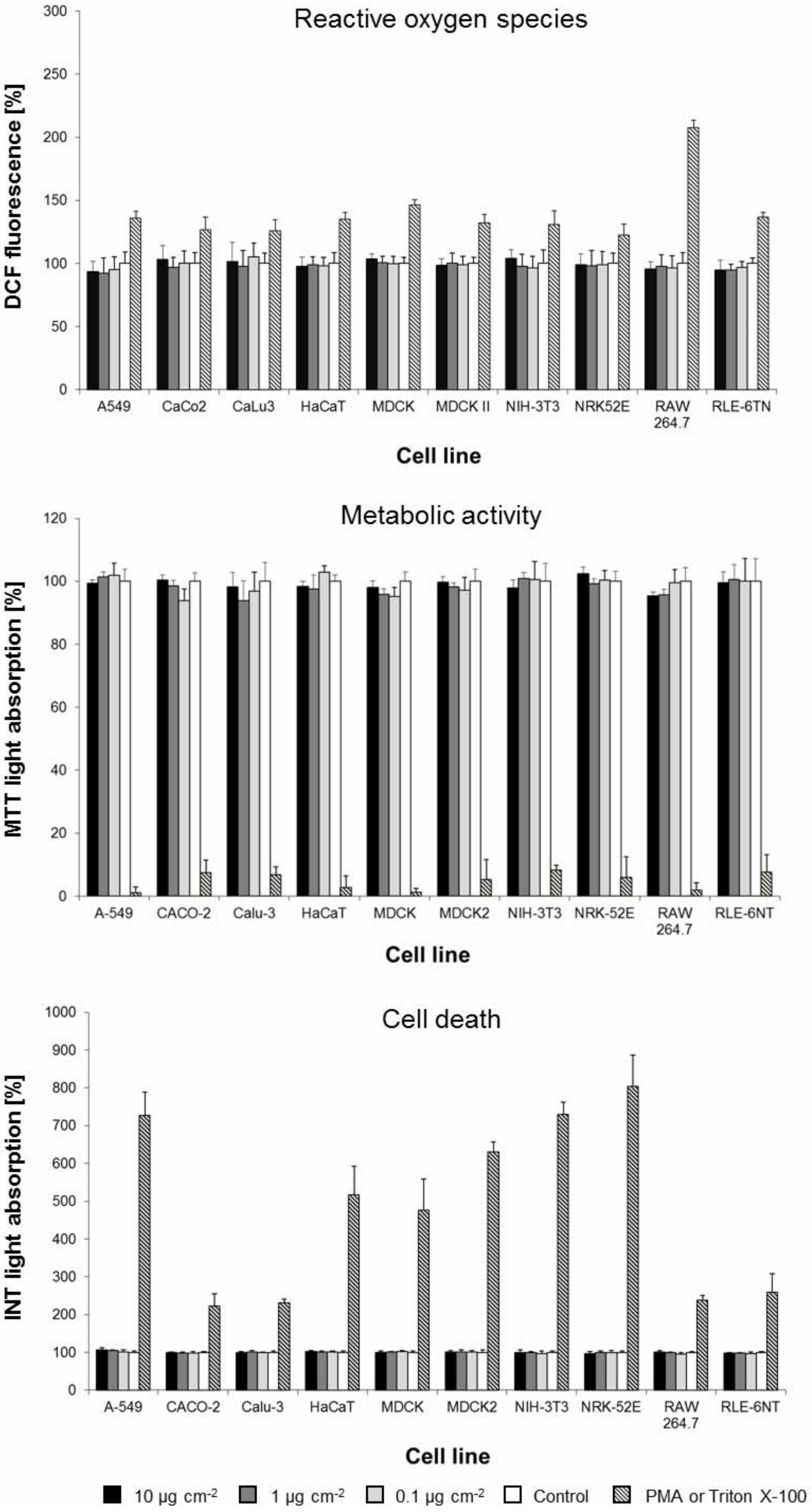

Reactive oxygen species
DCF fluorescence [%]
0
50
100
150
200
250
300
A549
CaCo2
CaLu3
HaCaT
MDCK
MDCK II
NIH-3T3
NRK52E
RAW 264.7
RLE-6TN
Cell line
Metabolic activity
MTT light absorption [%]
0
20
40
60
80
100
120
A-549
CACO-2
Calu-3
HaCaT
MDCK
MDCK2
NIH-3T3
NRK-52E
RAW 264.7
RLE-6NT
Cell line
Cell death
INT light absorption [%]
0
100
200
300
400
500
600
700
800
900
1000
A-549
CACO-2
Calu-3
HaCaT
MDCK
MDCK2
NIH-3T3
NRK-52E
RAW 264.7
RLE-6NT
Cell line
10 µg cm-2
1 µg cm-2
0.1 µg cm-2
Control
PMA or Triton X-100

S

ZrO_2 2

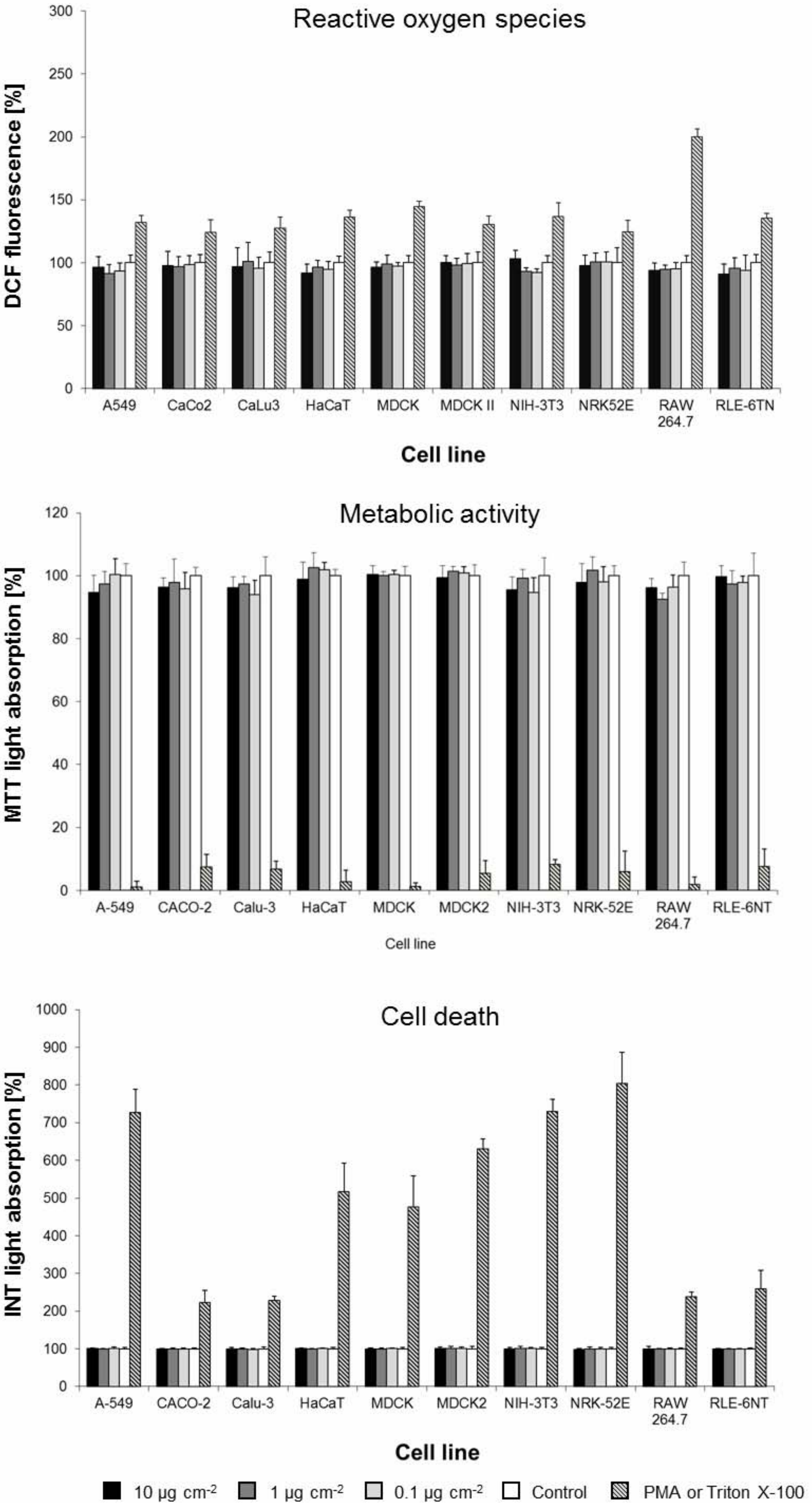
Reactive oxygen species
DCF fluorescence [%]
300
250
200
150
100
50
0
A549
CaCo2
CaLu3
HaCaT
MDCK
MDCK II
NIH-3T3
NRK52E
RAW 264.7
RLE-6TN
Cell line
Metabolic activity
MTT light absorption [%]
120
100
80
60
40
20
0
A-549
CACO-2
Calu-3
HaCaT
MDCK
MDCK2
NIH-3T3
NRK-52E
RAW 264.7
RLE-6NT
Cell line
Cell death
INT light absorption [%]
1000
900
800
700
600
500
400
300
200
100
0
A-549
CACO-2
Calu-3
HaCaT
MDCK
MDCK2
NIH-3T3
NRK-52E
RAW 264.7
RLE-6NT
Cell line
10 µg cm-2
1 µg cm-2
0.1 µg cm-2
Control
PMA or Triton X-100

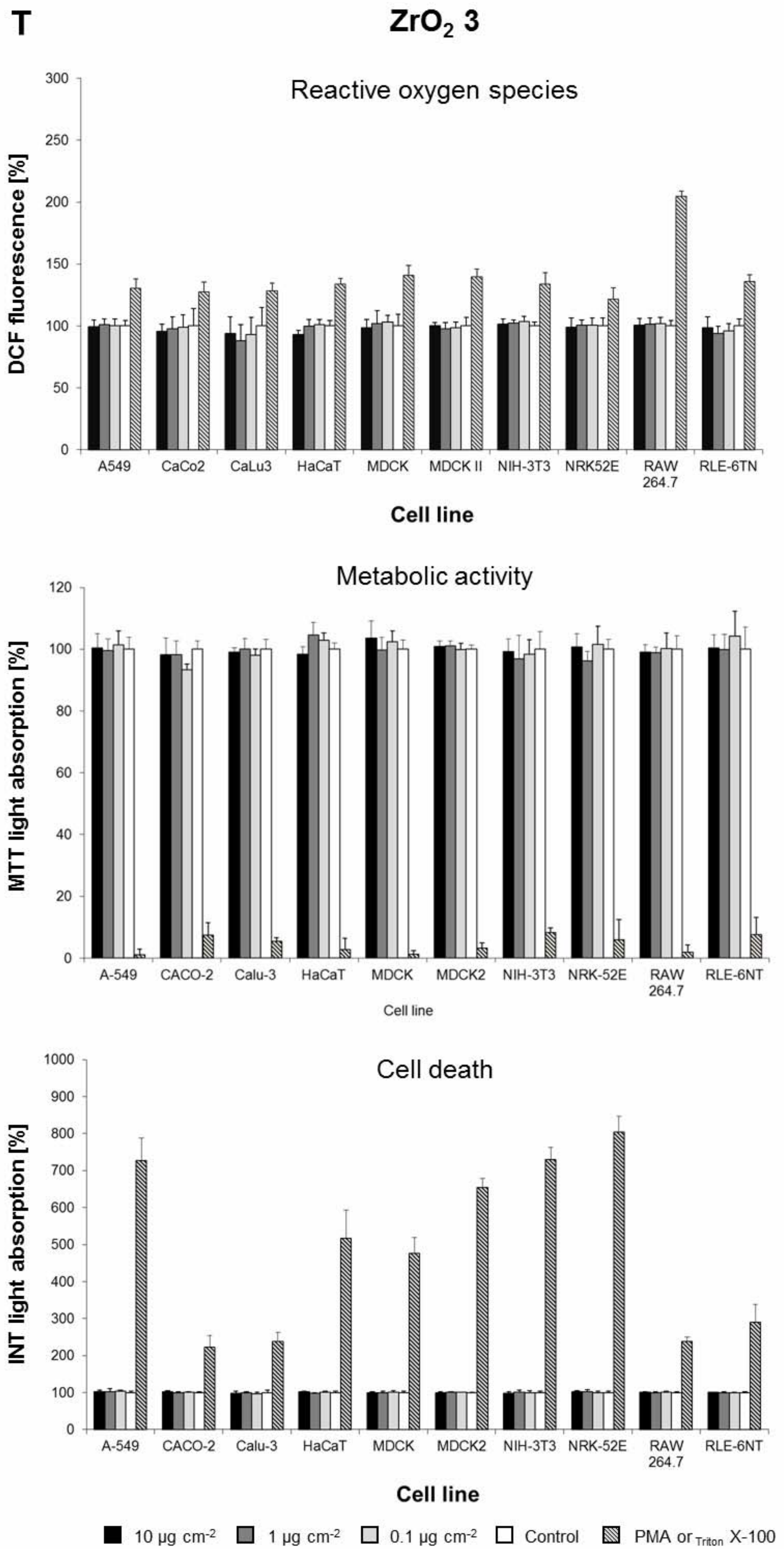
T
ZrO2 3
Reactive oxygen species
DCF fluorescence [%]
A549
CaCo2
CaLu3
HaCaT
MDCK
MDCK II
NIH-3T3
NRK52E
RAW 264.7
RLE-6TN
Cell line
Metabolic activity
MTT light absorption [%]
A-549
CACO-2
Calu-3
HaCaT
MDCK
MDCK2
NIH-3T3
NRK-52E
RAW 264.7
RLE-6NT
Cell line
Cell death
INT light absorption [%]
A-549
CACO-2
Calu-3
HaCaT
MDCK
MDCK2
NIH-3T3
NRK-52E
RAW 264.7
RLE-6NT
Cell line
10 µg cm-2
1 µg cm-2
0.1 µg cm-2
Control
PMA or Triton X-100

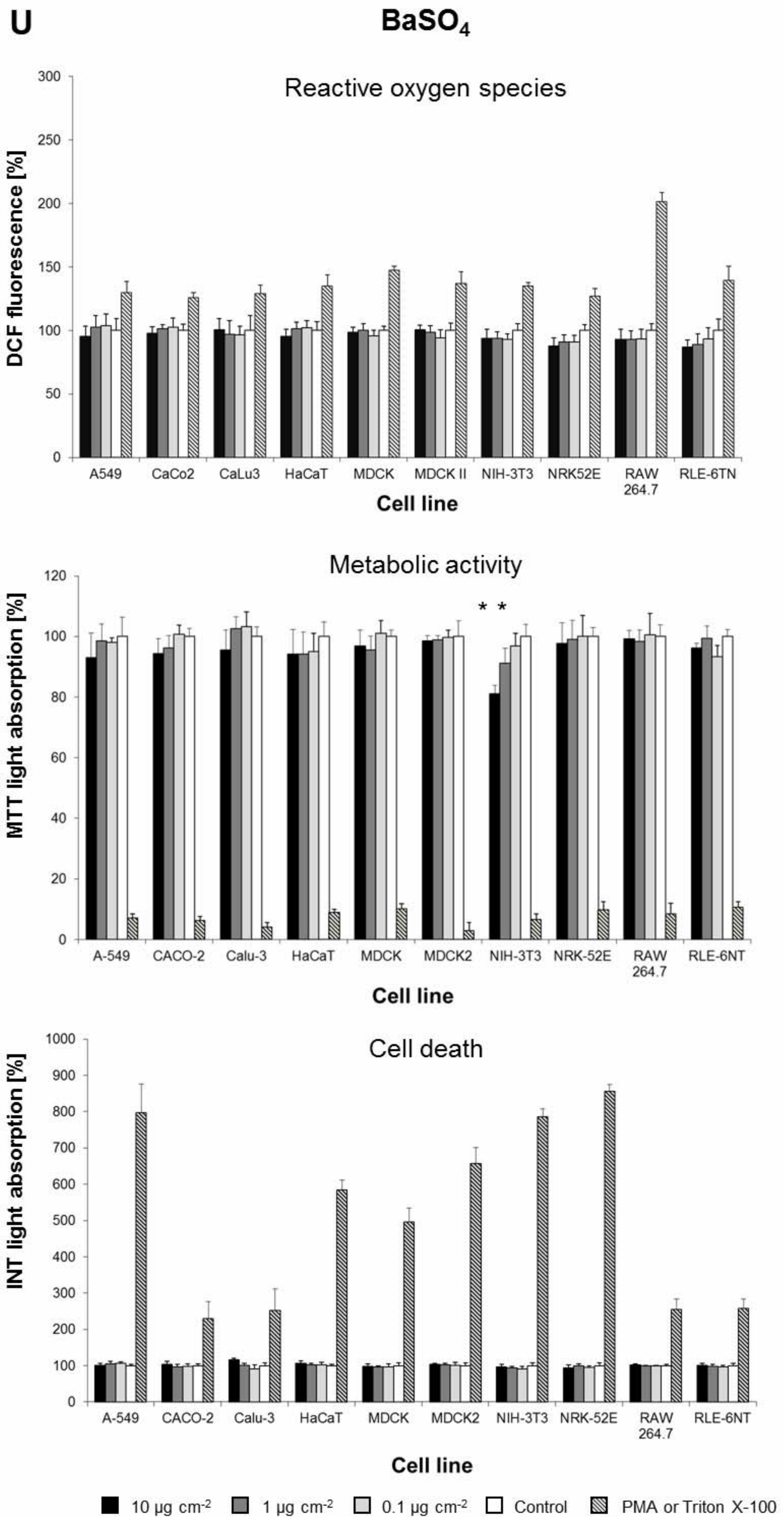
U
BaSO4
Reactive oxygen species
DCF fluorescence [%]
300
250
200
150
100
50
0
A549
CaCo2
CaLu3
HaCaT
MDCK
MDCK II
NIH-3T3
NRK52E
RAW 264.7
RLE-6TN
Cell line
Metabolic activity
MTT light absorption [%]
120
100
80
60
40
20
0
* *
A-549
CACO-2
Calu-3
HaCaT
MDCK
MDCK2
NIH-3T3
NRK-52E
RAW 264.7
RLE-6NT
Cell line
Cell death
INT light absorption [%]
1000
900
800
700
600
500
400
300
200
100
0
A-549
CACO-2
Calu-3
HaCaT
MDCK
MDCK2
NIH-3T3
NRK-52E
RAW 264.7
RLE-6NT
Cell line
10 µg cm-2
1 µg cm-2
0.1 µg cm-2
Control
PMA or Triton X-100

V SrCO$_3$ 1

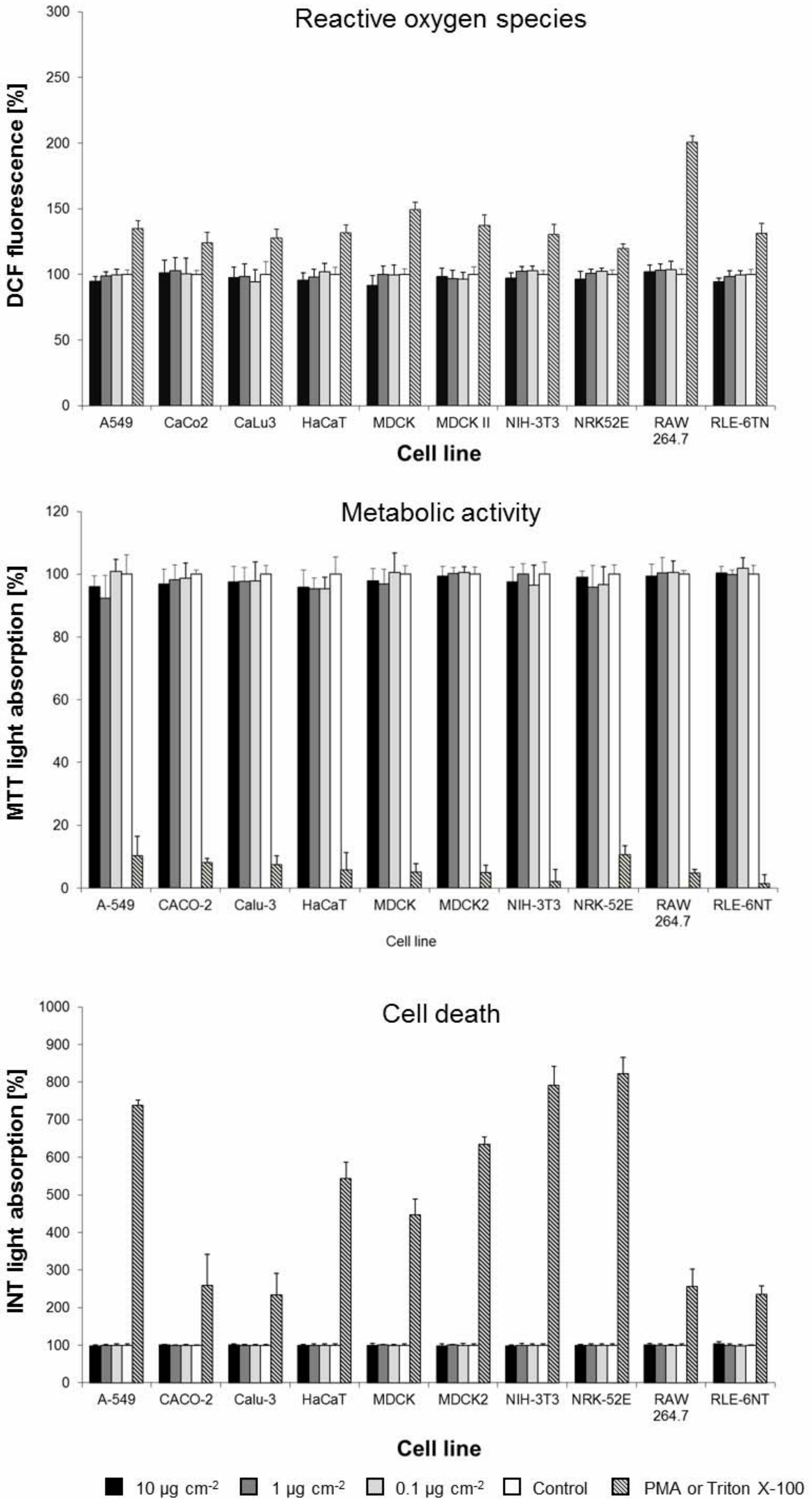

Reactive oxygen species
DCF fluorescence [%]
300
250
200
150
100
50
0
A549
CaCo2
CaLu3
HaCaT
MDCK
MDCK II
NIH-3T3
NRK52E
RAW 264.7
RLE-6TN
Cell line
Metabolic activity
MTT light absorption [%]
120
100
80
60
40
20
0
A-549
CACO-2
Calu-3
HaCaT
MDCK
MDCK2
NIH-3T3
NRK-52E
RAW 264.7
RLE-6NT
Cell line
Cell death
INT light absorption [%]
1000
900
800
700
600
500
400
300
200
100
0
A-549
CACO-2
Calu-3
HaCaT
MDCK
MDCK2
NIH-3T3
NRK-52E
RAW 264.7
RLE-6NT
Cell line
10 µg cm-2
1 µg cm-2
0.1 µg cm-2
Control
PMA or Triton X-100

W $SrCO_3$ 2

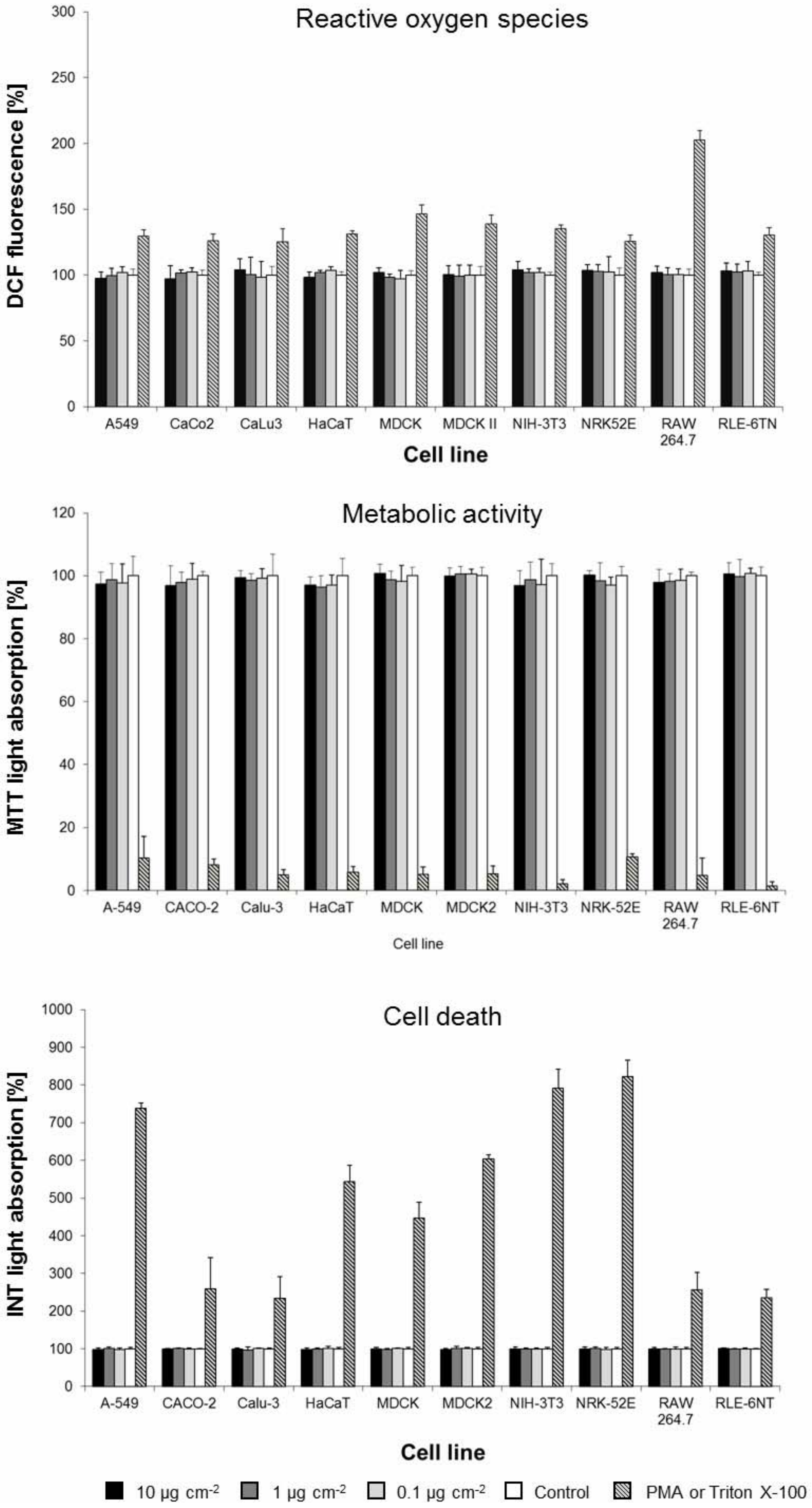
Reactive oxygen species
DCF fluorescence [%]
300
250
200
150
100
50
0
A549
CaCo2
CaLu3
HaCaT
MDCK
MDCK II
NIH-3T3
NRK52E
RAW 264.7
RLE-6TN
Cell line
Metabolic activity
MTT light absorption [%]
120
100
80
60
40
20
0
A-549
CACO-2
Calu-3
HaCaT
MDCK
MDCK2
NIH-3T3
NRK-52E
RAW 264.7
RLE-6NT
Cell line
Cell death
INT light absorption [%]
1000
900
800
700
600
500
400
300
200
100
0
A-549
CACO-2
Calu-3
HaCaT
MDCK
MDCK2
NIH-3T3
NRK-52E
RAW 264.7
RLE-6NT
Cell line
10 µg cm-2
1 µg cm-2
0.1 µg cm-2
Control
PMA or Triton X-100

Figure S3. ROS formation (DCF fluorescence), metabolic activity (MTT light absorption), and cell death (INT light absorption) in ten cell lines exposed to different concentrations of A TiO_2 1, B TiO_2 2, [TiO_2 3, Carbon Black, CeO_2 A, CeO_2 B, CeO_2 C, CeO_2 D], C CeO_2, D AlOOH I, E AlOOH II, F Ti-Zr1, G Ti-Zr 2, [Ti-Zr 3], H Al-Ti-Zr 1, I Al-Ti-Zr 2, J Al-Ti-Zr 3, K ZrO_2 1, L ZrO_2 2, M ZrO_2 3, [$BaSO_4$], N $SrCO_3$ 1, O $SrCO_3$ 2 in DMEM / 10 % FBS or pure DMEM / 10 % FBS (Control) Data are expressed as % of control mean ± SD of three independent experiments with seven replications each.

Characterization of cell density and adhesion by Immunofluorescence Microscopy

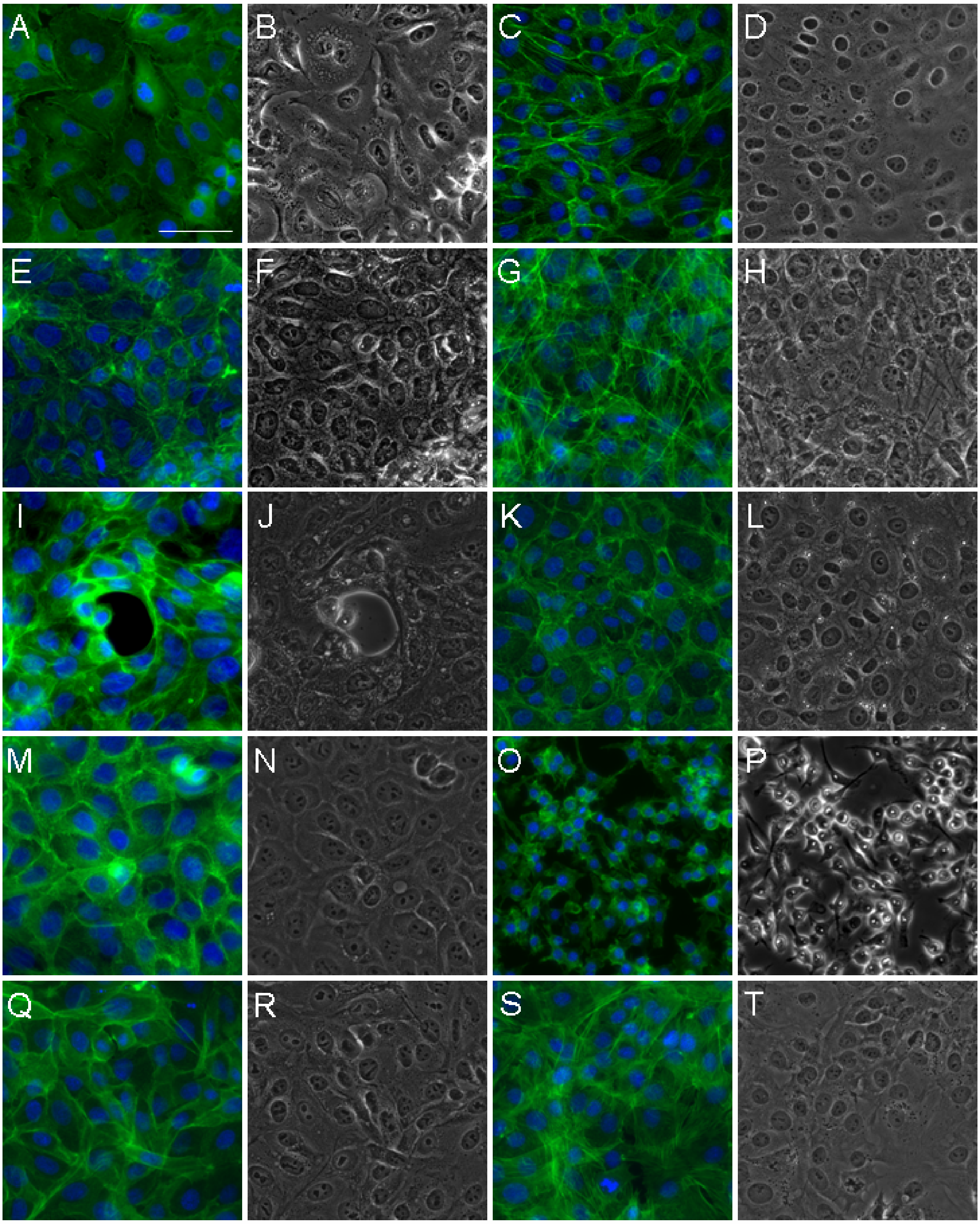

Figure S4. Fluorescence and phase contrast images of ten different cell lines. Cells were cultured on coverslips, fixated after 24 h, filamentous actin (FITC filter system, exposure time 1500ms) and nuclei (DAPI filter system, exposure time 50ms) were stained, respectively. A549 (A, B); MDCKII (C, D); CaCo2 (E, F); NIH-3T3 (G, H); CaLu-3 (I, J); NRK-52E (K, L); HaCaT (M, N); RAW264.7 (O, P); MDCK (Q, R); RLE-6TN (S, T). Images were background-corrected. Bar: 50 µm
